# scHG: A supercell framework with high-order graph learning enables scalable multi-omics analysis

**DOI:** 10.1371/journal.pcbi.1013851

**Published:** 2026-05-06

**Authors:** Yixiang Huang, Yuan Gan, Xinqi Gong

**Affiliations:** School of Mathematics, Renmin University of China, Beijing, China; Guangxi University, CHINA

## Abstract

Multi-omics profiling—spanning proteomics, transcriptomics, and additional omics data types—is rapidly advancing, providing increasingly detailed maps of cellular identity and function. Yet, identifying rare cell populations while maintaining computational tractability remains a major challenge in large-scale multi-omics clustering. Here, we introduce the *supercell* paradigm, in which expression-coherent cells are grouped into intermediate units that preserve weak but biologically meaningful local structure across omics layers, thereby improving sensitivity to rare populations that are often masked at the conventional cluster level. Supercells are constructed using angle-aware similarity metrics and second-order co-occurrence neighbors, with impurity cells pruned by degree centrality. Building on this idea, we develop scHG, a high-order graph learning framework with an omics-weighted optimizer that adaptively balances contributions from gene expression, surface proteins, and chromatin accessibility while remaining scalable on large datasets through sparse matrix optimization and iterative graph refinement. Across six benchmark datasets (up to 30672 cells), scHG consistently outperforms state-of-the-art methods, improving mean ARI and NMI by 3.97% and 3.54%, respectively, while reducing runtime by 26.40%. Beyond overall clustering accuracy, scHG resolves fine-grained heterogeneity within conventionally defined T-cell populations and, importantly, uncovers rare populations—including dendritic-cell populations and NK-like B cells—that remain hidden under standard clustering pipelines. These results demonstrate that supercells provide not only an efficient intermediate representation for large-scale multi-omics integration, but also a practical mechanism for rare-cell detection.

## Author summary

Modern single-cell technologies can measure multiple molecular layers from the same cell, such as RNA, surface proteins, and chromatin accessibility. These rich “multi-omics” profiles promise a more complete view of cellular identity, but they also create a practical bottleneck: existing methods can be slow on large datasets and often miss rare yet important cell populations. We present scHG, a fast and accurate framework that compresses many similar cells into intermediate units called “supercells” and then learns relationships among supercells using a high-order graph model. This design keeps biologically meaningful structure while dramatically reducing computational cost, making large-scale analyses feasible on standard hardware. In benchmarks spanning multiple multi-omics datasets, scHG improves clustering accuracy and runs substantially faster than state-of-the-art approaches. Beyond overall performance, scHG reveals fine-grained immune subtypes within T cells and highlights rare populations—such as dendritic cells and NK-like B cells—that are easily diluted in conventional cluster-level analysis. By combining efficiency with sensitivity to subtle and rare signals, scHG helps researchers map cellular diversity more reliably in complex multi-omics studies.

## 1 Introduction

Thanks to the rapid development of bioinformatics tools, multi-omics analysis has flourished in recent years. As a fundamental task, clustering provides the foundation for a wide range of downstream analyses, including cellular heterogeneity characterization, analysis of cell development trajectory, and cell-cell communication inference. Given that different omics modalities—such as the proteomics, transcriptomics, and chromatin accessibility—capture complementary aspects of cellular states, an essential challenge is how to effectively integrate these heterogeneous datasets for joint clustering. This integration problem is naturally formulated as a multi-view clustering task, where each omics modality represents a distinct view of the same underlying biological system.

Multi-view clustering has long been studied in machine learning, with representative strategies including latent-variable modeling, canonical-correlation-based integration, matrix factorization, spectral clustering, and adaptive view weighting [1–9]. These methods provide important foundations for integrating heterogeneous observations from multiple views, but most were developed for general settings rather than the sparsity, noise, and scale of single-cell multi-omics data.

These ideas inspired early multi-omics clustering methods in bioinformatics, especially matrix-factorization and latent-variable models such as scAI, LIGER, BREM-SC, scMNMF, and GSTRPCA [10–14]. Such methods have substantially advanced joint analysis across omics layers, but their ability to capture complex higher-order cell-cell structure remains limited, especially when rare populations are weakly separated from dominant cell groups.

In recent years, graph-based approaches have emerged as a competitive paradigm for multi-view clustering, particularly suited to the complexity and noise inherent in omics data. By leveraging relational information both within and across views, these methods mitigate the shortcomings of earlier factorization-based techniques, offering improved structural fidelity and resilience to outliers. Representative advances include GBS [15], which established a general graph-based framework for multi-view learning, and CGD [16], which employed cross-view graph diffusion to strengthen inter-view communication. Methods such as GMC [17] and Consensus Graph Learning [18] further emphasized the construction of unified or consensus graphs that distill view-shared structures. In the bioinformatics setting, Jiang et al. [19] introduced a Laplacian optimization framework for robust clustering of multi-omics data, demonstrating the adaptability of graph-based formulations for encoding feature dependencies. These graph-based methods exhibit *O*(*n*^3^) complexity, primarily due to the eigen-decomposition required in spectral clustering [15,17–19] and the large-scale matrix multiplications involved [16]. Taken together, these developments consolidate graph-based models as a robust and versatile framework for multi-view integration and clustering across both machine learning and biological domains.

However, despite their partial effectiveness, current graph-based methods still face two major challenges that limit their scalability and resolution on large-scale multi-omics datasets. First, since eigen-decomposition in spectral clustering or large-scale matrix multiplications, graph-based methods generally incur a computational cost of *O*(*n*^3^), making them infeasible for large-scale omics datasets. Second, rare cell populations remain difficult to detect, since separating small yet expression-coherent groups without disrupting global cluster integrity requires capturing subtle higher-order relationships. This calls for further exploration of distance metrics and refined representations of cell-to-cell similarity that go beyond traditional pairwise measures. Together, these challenges underscore the need for frameworks that can efficiently incorporate higher-order structure while maintaining computational tractability.

**Fig 1:**
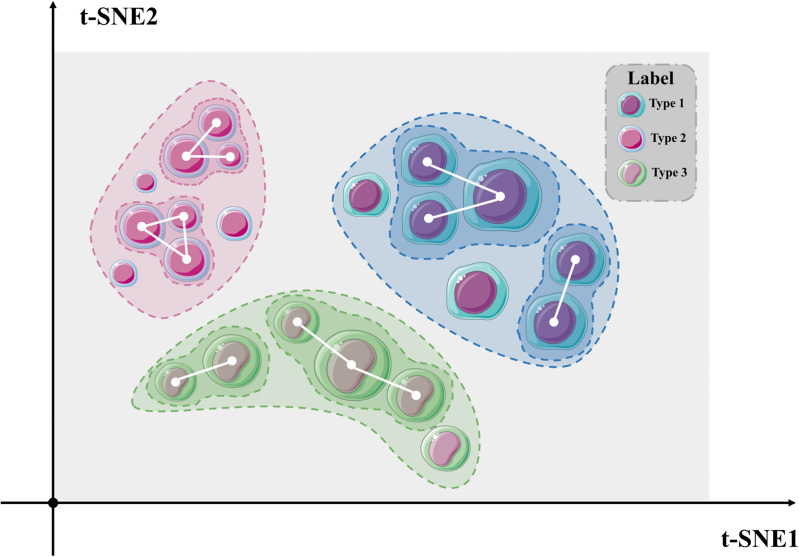
Visualization of t-SNE based on supercell clustering results.

To overcome these limitations, we propose a novel high-order neighbor-aware coarse-grained multi-omics graph clustering framework. The core innovation of our approach lies in the introduction of the supercell concept (Box 1), which aggregates cells into biologically meaningful units when their expression profiles display higher-order consistency that is stably preserved across multiple omics layers (Fig 1). These units operate at an intermediate resolution between single cells and tissue-scale structures, providing a principled means to balance fine-grained heterogeneity with computational efficiency. The formulation of supercells serves two primary objectives. First, by aggregating cells into coherent units, we dramatically reduce the effective sample size, thereby enhancing scalability and rendering our framework suitable for large-scale datasets. Second, supercells enable the capture of more subtle cellular subtypes without increasing the number of clusters, thereby facilitating the identification of rare cell populations.

Intermediate-resolution representations have been explored in multiple contexts, including metacell-based compression (MetaCell [20]), pseudobulk-style aggregation for robust testing, archetype/landmark constructions (SEACells [21]), and graph abstractions for trajectory discovery (PAGA [22]). However, these approaches typically define intermediate units using single-omic similarity or task-specific heuristics and do not explicitly enforce cross-omics neighborhood concordance as a defining criterion of the unit itself. In contrast, our supercells are constructed as connected components on a fused multi-omics consistency graph derived from second-order co-occurrence neighbors, so that each supercell represents a set of cells whose local neighborhoods are persistently shared across modalities. Moreover, we introduce a degree-centrality–based probabilistic pruning procedure to remove low-coherence boundary or outlier cells within each candidate component, thereby improving supercell purity before downstream clustering.

**Box 1. What is a supercell?**

In this study, we use the term **supercell** consistently to denote the intermediate-resolution unit constructed by scHG. A supercell is defined as a group of cells whose local neighborhoods remain coherent across multiple omics modalities and that are retained after degree-centrality–based probabilistic pruning. Unlike a conventional cluster, which represents a final partition of the dataset, a supercell is an intermediate structured learning unit used to preserve local topology, improve scalability, and enhance sensitivity to rare or weakly separated populations.

As a result, the supercell in scHG is intended not merely as an aggregated summary for compression, but as a structured learning unit that preserves modality-consistent local topology for downstream high-order graph representation learning. This distinction is particularly important for rare-population discovery, where biologically meaningful signals are often vulnerable to dilution under generic aggregation schemes; by contrast, the fused neighborhood-consistency construction and probabilistic pruning in scHG help retain weak yet coherent cross-omics structures. In this way, the resulting intermediate units serve not only as a device for scalability, but also as a topology-aware graph substrate for subtype-aware and rare-population-sensitive multi-omics clustering (Table 1).

**Table 1:** Comparison of intermediate-resolution approaches. Columns indicate whether a method explicitly incorporates each design property.

Approach	Explicit supercell/landmark construction	Multi-omics concordance	High-order neighborhood	Outlier/impurity handling	Graph learning on units
**MetaCell** [20]	✓	–	–	✓	✓
**Squair** [23]	✓	–	–	✓	–
**SEACells** [21]	✓	–	✓	✓	✓
**PAGA** [22]	✓	–	✓	✓	✓
**scHG**	✓	✓	✓	✓	✓

Our study makes three main contributions. First, we introduce the supercell concept into multi-omics clustering and show that it can reveal rare populations, such as cDC2-like and NK-like B-cell-related signals, that are masked at the conventional cluster level. Second, we build a scalable graph-based multi-omics clustering framework that performs strongly across six benchmarks, while clustering datasets with more than 30,000 cells in under 20 minutes. Third, we develop a high-order graph model that combines second-order co-occurrence neighbors, angle-aware similarity, and probabilistic pruning to improve supercell purity and clustering robustness while adaptively estimating cluster numbers.

## 2 Results

### 2.1 Overview of scHG

**Fig 2:**
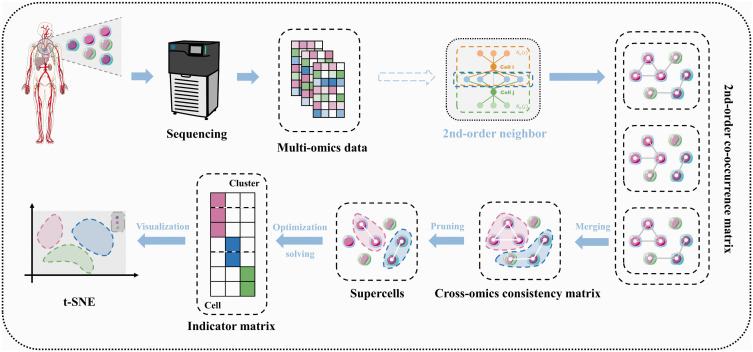
The workflow includes: (1) second-order co-occurrence neighbor extraction from multi-omics data; (2) cross-omics consistency fusion; (3) probabilistic pruning to identify high-order structural units (supercells); (4) supercell clustering via optimization; and (5) visualization and downstream analyses.

Our workflow delivers a two-stage, graph-based partitioning that first groups cells into supercells and then organises those supercells into higher-level clusters (Fig 2).

**Stage 1: Supercell formation.** For each omics layer, we build a γ-nearest-neighbor graph with Pearson-correlation weights, promote it to a second-order graph by requiring mutual α - neighborhood and at least β shared neighbors, and then fuse all layers into a cross-omics consistency graph. Connected components in this fused graph form supercell candidates; degree - centrality – based pruning removes low-coherence outliers, yielding biologically coherent supercells that retain both local co-expression and global manifold structure.

**Stage 2: Supercell clustering.** The final optimized model formulation is given by Eq. (1) (see Section 7.2 for derivation):


$$\begin{array}{c} \begin{array}{c} \min_{{𝐄}_{S\times C},\{\omega \left(v\right){\}}_{v=1}^{V}}\sum_{v=1}^{V}\omega \left(v\right)\sum_{p=1}^{C}\|𝐋\left(v\right){𝐞}_{p}{\|}_{1}\;s.t.\sum_{v=1}^{V}\frac{1}{\omega \left(v\right)}=1,\{\omega \left(v\right){\}}_{v=1}^{V}⪰0,\;𝐄\in \{0,1{\}}_{S\times C},E1=1, \end{array} \end{array}$$ (1)

where ${𝐄}_{S\times C}=(e_{1},e_{2},...,e_{C})$ denotes the clustering result of supercells, $\{\omega \left(v\right){\}}_{v=1}^{V}$ represents the optimization weights for distinct omics layers, 𝐋(v) is the Laplacian matrix of $AS\left(v\right)=(as_{yz}\left(v\right))$ defined in Eq. (17).

We iteratively optimize cluster labels and modality weights with block-coordinate descent. In each iteration, omics-specific Laplacian matrices are used to update modality weights, and a composite Laplacian is then used to assign supercells to clusters. This procedure yields a modality-weighted partition that balances cross-omic consensus and cluster separation. A more detailed derivation is provided in Section 7.

### 2.2 scHG enhances the resolution of cell subpopulation recognition

For the clustering results obtained by scHG on the PBMC10× dataset, we performed one-sided Welch’s *t*-tests (Null hypothesis $H_{0}:\mu_{\text{Target Cluster}}\leq \mu_{\text{Others}}$; al*t*ernative hypothesis $H_{1}:\mu_{\text{Target Cluster}}>\mu_{\text{Others}}$) to identify enriched markers across clusters and subsequently assigned cell-type labels. Specifically, for each predicted cluster, we compared cells within the cluster against all remaining cells using Welch’s two-sample *t*-test with unequal variances. We reported one-sided evidence for up-regulation in the target cluster, retaining only features wi*t*h higher mean expression in the cluster. To directly address multiple comparisons, we additionally applied Benjamini–Hochberg correction across all tested genes/proteins within each cluster and computed FDR-adjusted one-sided *p*-values; notably, all reported markers remain significant after correction (FDR < 0.05). Marker candidates were selected using a joint criterion of *p* < 0.05 and $\log_{2}FC>0.25$, where


$$\begin{array}{c} \begin{array}{c} \log_{2}FC=\log_{2}({\bar{x}}_{cluster}+\epsilon )-\log_{2}({\bar{x}}_{rest}+\epsilon ) \end{array} \end{array}$$ (2)

with $\epsilon =10^{-6}$. Notably, Clusters 3 and 7 shared a core set of T-cell markers—CD3, CD4, CD25, CD45RO, PD-1 and CD127—indicating that both represent CD4^+^ T-cell populations (Table 2).

**Table 2:** Top enriched markers in cluster 3 and cluster 7 based on one-sided Welch’s t-test.

Cluster	Marker	Type	p-value
Cluster 3	CD3_TotalSeqB	Protein	$<1\times 10^{-300}$
	CD4_TotalSeqB	Protein	$<1\times 10^{-300}$
	CD127_TotalSeqB	Protein	$2.27\times 10^{-204}$
	CD45RO_TotalSeqB	Protein	$3.14\times 10^{-58}$
	PD-1_TotalSeqB	Protein	$1.41\times 10^{-33}$
	CD25_TotalSeqB	Protein	$2.95\times 10^{-32}$
	CD27	RNA	$8.48\times 10^{-67}$
	HSPA8	RNA	$1.26\times 10^{-23}$
	AQP3	RNA	$1.04\times 10^{-21}$
	PIM2	RNA	$2.36\times 10^{-18}$
Cluster 7	CD16_TotalSeqB	Protein	$9.87\times 10^{-21}$
	CD4_TotalSeqB	Protein	$3.79\times 10^{-17}$
	CD15_TotalSeqB	Protein	$1.44\times 10^{-14}$
	CD45RO_TotalSeqB	Protein	$3.15\times 10^{-9}$
	CD3_TotalSeqB	Protein	$1.50\times 10^{-8}$
	CD127_TotalSeqB	Protein	$1.61\times 10^{-6}$
	CD25_TotalSeqB	Protein	$2.66\times 10^{-4}$
	PD-1_TotalSeqB	Protein	$6.71\times 10^{-4}$
	TIGIT_TotalSeqB	Protein	$3.38\times 10^{-2}$
	CCND2	RNA	$2.11\times 10^{-3}$

Beyond this common signature, however, each cluster exhibited distinct molecular programs. Cluster 3 was distinguished by transcriptional enrichment of activation- and proliferation-related genes, including *CD27*, *PIM2* and *HSPA8*, together with a broad set of cell-cycle regulators such as *MKI67*, *CCNB1*, *EZH2*, *CHEK1* and members of the *MCM* family. This molecular profile points to a stem-like or central-memory T-cell subpopulation with features of early exhaustion, consistent with previous observations [24,25].

By contrast, Cluster 7 displayed a markedly distinct surface phenotype, with significant enrichment of CD16, CD15 and TIGIT in addition to the shared T-cell markers. The co-expression of CD16 and CD15—typically associated with NK cells and granulocytes, respectively—points to the emergence of an innate-like cytotoxic T-cell subpopulation. Such CD16^+^ T cells have been recognized as functionally distinct subpopulations with heightened cytolytic potential [26,27], while CD15 expression, though rare, has been observed in activated γδ T cells and NKT-cell subpopulations [28].

Taken together, these results show that scHG resolves biologically distinct subpopulations within the broader CD4^+^ T-cell compartment, highlighting its ability to increase subpopulation resolution without using prior labels.

### 2.3 Label-free identification of clinically significant B-cell biomarkers

To assess the biological validity of our framework, we examined Cluster 5 (S1 Table), which is labelled as a B-cell population by the reference annotations. Applying a one-sided Welch’s *t*-test, we identified a panel of markers that are significantly enriched in both the transcriptomic and proteomic modalities.

At the protein modality, classical B-cell surface markers— CD19 ($p=2.03\times 10^{-168}$) and CD45RA ($p=2.47\times 10^{-118}$)—were markedly enriched in Cluster 5 [29]. At the transcriptomic modality, we observed significant up-regulation of canonical B-cell genes, including *CD74* ($p=1.43\times 10^{-158}$) [30], *MS4A1* ($p=4.08\times 10^{-104}$) [31], *BANK1* ($p=5.89\times 10^{-101}$) [32,33], and *CD79A* [34]. Moreover, multiple MHC class II genes—*HLADQB1*, *HLADQA1*, and *HLADRB1*—were strongly over-expressed ($p<1\times 10^{-50}$) [35], collectively corroborating the B-cell identity of this cluster.

Importantly, these markers are not only canonical B-cell identifiers but also represent validated or emerging therapeutic targets. For instance, CD19 is one of the most prominent B-cell antigens exploited in contemporary immunotherapy, underpinning multiple CAR-T products and monoclonal antibodies [36]. *MS4A1* constitutes the molecular target of rituximab and next-generation anti-CD20 antibodies, which are widely deployed in the treatment of B-cell lymphomas and autoimmune diseases [31]. *CD79A*, an essential component of the B-cell receptor (BCR) complex, has likewise been proposed as a druggable node for suppressing aberrant BCR signalling [37]. Finally, *CD74*, which functions in MHC class II antigen presentation and serves as a receptor for macrophage migration inhibitory factor (MIF), is currently being pursued with small-molecule inhibitors in pre-clinical studies [38].

Overall, these results show that scHG can recover biologically meaningful and clinically relevant marker programs in a label-free manner.

### 2.4 Cross-omics enrichment validates scHG in uncovering coordinated NK-cell effector programs

To further elucidate the functional implications of the markers up-regulated in the clusters identified by scHG, we conducted Gene Ontology (GO) enrichment analysis at both the transcriptomic and proteomic levels (S2 Table).

**Fig 3:**
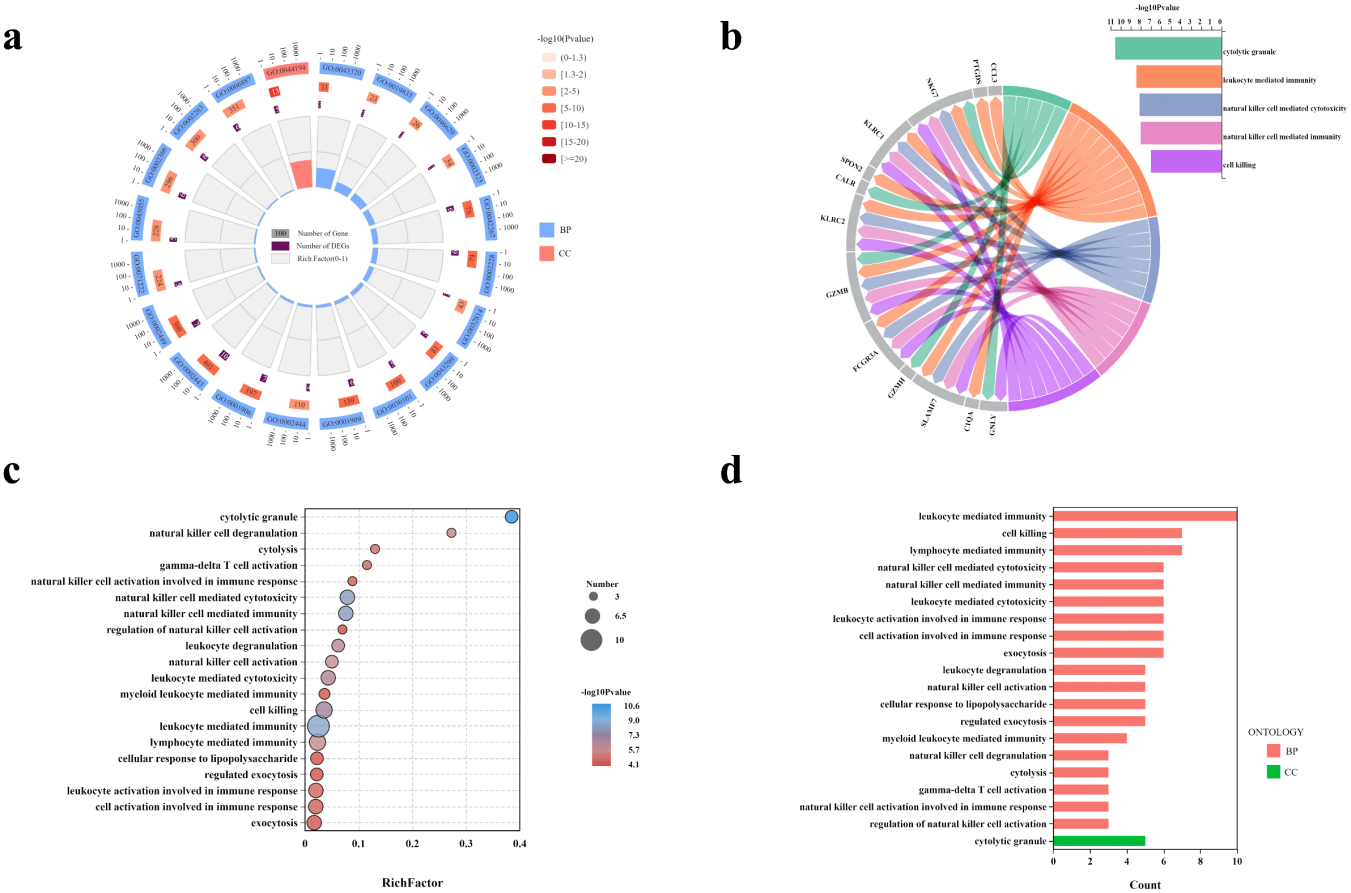
Functional enrichment analysis of upregulated genes. **(a)** GO circular plot showing total and differentially expressed genes (DEGs) associated with each GO term. **(b)** Chord diagram linking representative upregulated genes to enriched immune-related processes. **(c)** GO enrichment bubble plot. **(d)** Bar plot of significantly enriched GO terms.

We analysed Cluster 2, which is annotated as a natural-killer (NK) cell population. At the transcriptomic level, as depicted in Fig 3, the bar plot (Fig 3d) reveals that the most significantly enriched Gene Ontology (GO) terms—including *leukocyte-mediated immunity*, *cell killing*, and *natural killer cell mediated cytotoxicity*—mirror the cytotoxic immune function of this cluster. The bubble plot (Fig 3c) shows that pathways related to NK-cell activation, immune regulation, and degranulation score highest, with terms such as *cytolytic granule* achieving the greatest statistical significance. The circular plot (Fig 3a) contrasts the total and differentially expressed genes (DEGs) associated with each GO term, underscoring the pronounced enrichment in immune-related pathways. The chord diagram (Fig 3b) maps key up-regulated genes to representative biological processes, showing that *GZMB*, *NKG7*, and several *KLR* family members are pivotal mediators of cell-killing and NK-cell immune responses.

**Fig 4:**
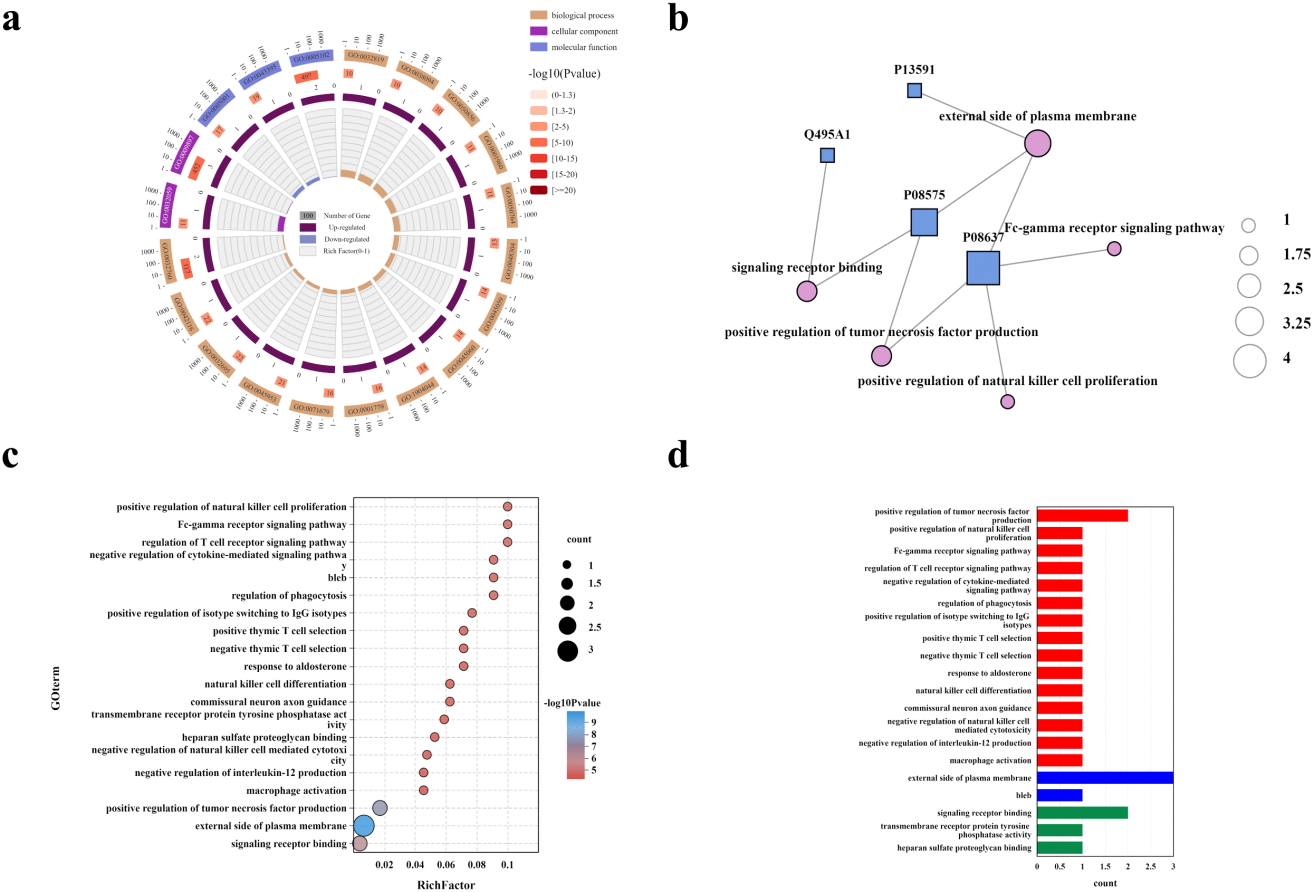
GO enrichment analysis of upregulated protein-level markers. **(a)** Circular GO plot summarizing enriched terms. **(b)** Network linking representative proteins to enriched GO terms. **(c)** Bubble plot of top enriched GO terms. **(d)** Bar plot of significantly enriched GO terms.

At the proteomic level, the enrichment pattern is consistent with the transcriptomic signal and further supports an activated NK-cell program (Fig 4). The dominant GO terms are related to cell-surface localization, receptor binding, and cytokine-associated immune activation, including positive regulation of tumor necrosis factor production and positive regulation of natural-killer-cell-mediated cytotoxicity. Together, the transcriptomic and proteomic analyses support a coherent cross-omics effector program centered on NK-cell activation, membrane-associated signaling, and cytotoxic function.

### 2.5 Supercell reveals rare cell populations

The supercell framework aggregates cells with coherent expression profiles into compact units. Supercells comprising a large number of cells are more likely to display distinct expression patterns relative to the surrounding cluster, thereby facilitating the identification of rare cell populations. We illustrate this principle with two examples.

In the PBMC10× dataset, one-sided Welch’s *t*-tests revealed tha*t* Cluster 6 was enriched for CD3, CD8A, CD8B, GZMK/GZMH/NKG7, CD45RO, PD-1, TIGIT, and CD127, consistent with a CD8^+^ T-cell phenotype. Intriguingly, several dendritic-cell markers—*CD1C*, *CD1E*, *CLEC10A*, *FCER1A*, and *CD74*—were also detected, suggesting the presence of an antigen-presenting cell subset. To disentangle this heterogeneity, we separately analysed Supercell 62—the largest unit within Cluster 6—and Cluster 6 with Supercell 62 excluded. Examined in isolation, Supercell 62 displayed a coherent transcriptional programme characteristic of conventional dendritic cells (cDC2). It showed strong up-regulation of classical DC markers (*CD1C*, *CD1E*, *FCER1A*, *CLEC10A*) together with MHC class II genes (*HLA-DPA1*, *HLA-DQA1*, *HLA-DPB1*, *CD74*). Strikingly, these genes were not significantly enriched when Cluster 6 was considered as a whole, indicating that their signal was largely masked at the cluster level. Additional genes—including *MS4A6A*, *IRF4*, *NDRG2*, and *LINC00926*—were likewise elevated only in Supercell 62, further substantiating its distinct identity and antigen-presenting potential. When Supercell 62 was excluded from Cluster 6, all dendritic-cell markers lost statistical significance, and the residual cluster reverted to a CD8^+^ T-cell–like or otherwise heterogeneous phenotype. This analysis demonstrates that the apparent cDC2-like programme within Cluster 6 was driven almost entirely by Supercell 62. To assess whether this type of signal could also be observed beyond the primary PBMC10× dataset, we examined an independent PBMC dataset (PBMC_Inhouse) at the same mesoscopic resolution. In that dataset, we identified a small subgroup with a dendritic-like antigen-presenting profile, characterized by CD11c at the protein level together with transcriptomic enrichment of *SPI1*, *FCN1*, *MNDA*, *LGALS1*, *TNF*, and *NFKBIA* (S6 Table). Although this subgroup did not exhibit the full cDC2 marker set observed in Supercell 62, it is nevertheless consistent with an APC-like myeloid state and provides external support for the broader conclusion that supercell-level analysis can recover small antigen-presenting populations that are diluted at conventional cluster resolution.

**Fig 5:**
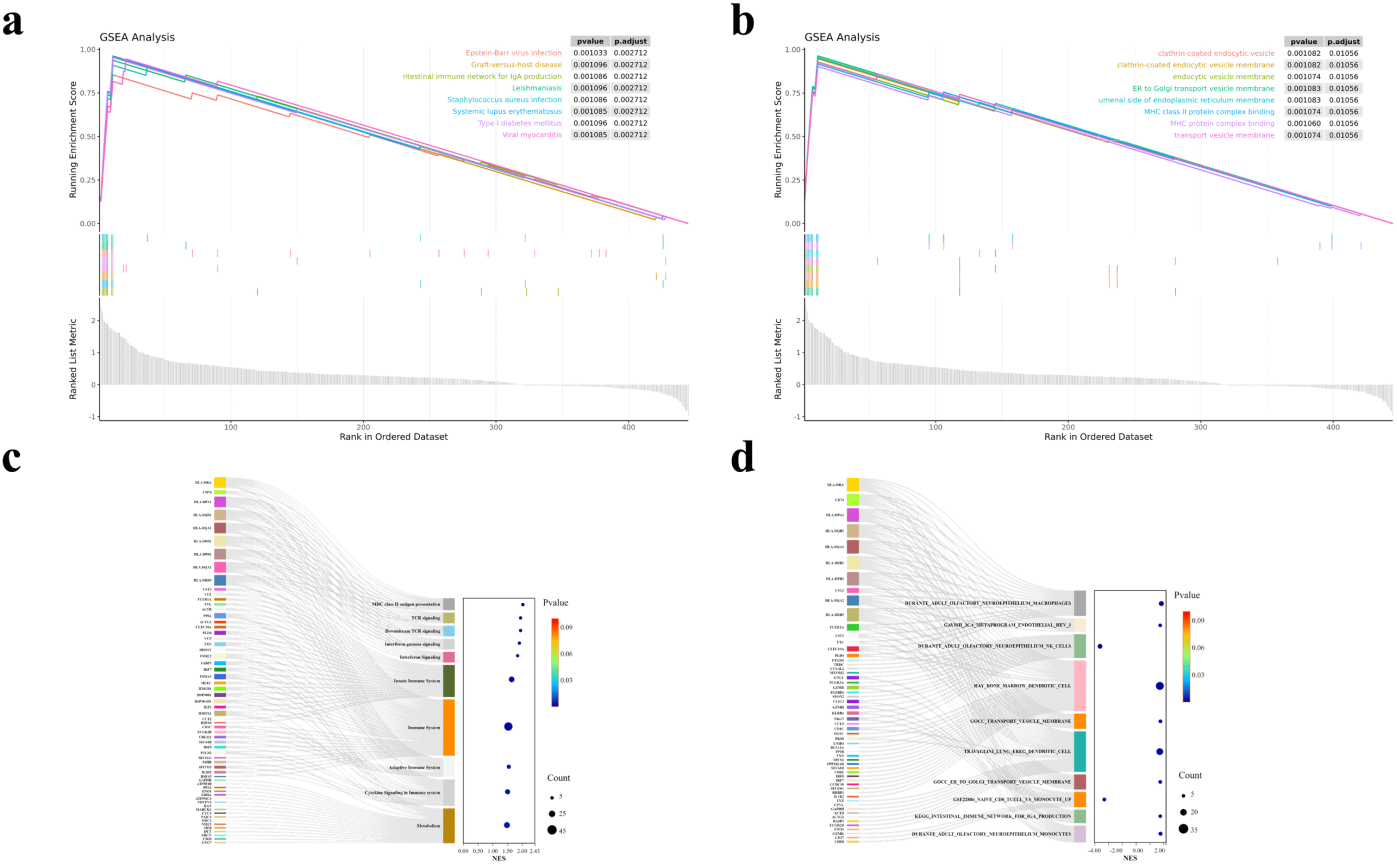
Pathway enrichment analysis for Supercell 62 across four databases. **(a)** KEGG pathway enrichment. **(b)** GO component enrichment. **(c)** Reactome pathway enrichment. **(d)** MSigDB enrichment. The results consistently highlight antigen presentation, dendritic cell programs, vesicle trafficking, and immune signaling pathways.

To further characterize Supercell 62, we performed gene-set enrichment analysis across GO, Reactome, MSigDB, and KEGG (S3 Table; Fig 5). Across all four resources, the enriched programs consistently pointed to antigen presentation, vesicle trafficking, and dendritic-cell-associated immune functions, supporting the interpretation of Supercell 62 as a cDC2-like population whose signal is diluted at the full-cluster level.

Another instance of a rare cell population revealed by supercell modelling is exemplified by Supercell 363, the largest mesoscopic unit embedded within Cluster 5, which is annotated as a B-cell population (S4 Table). A comparative analysis—Supercell 363 and Cluster 5 with Supercell 363 excluded—showed that the NK-cell–associated surface proteins CD56 and TIGIT were significantly up-regulated only in Supercell 363. These signals were diluted in the full cluster and were lost once Supercell 363 was excluded, indicating that NK-related expression is confined to this supercell unit and masked at the bulk-cluster level. The coexistence of both NK and B cell markers within a single supercell suggests a rare composite phenotype, which has been previously reported as rare natural killer-like B cells (NKB) [39]. This interpretation is further supported by the positional and annotation context of Supercell 363: it remains embedded within a B-cell-annotated parent cluster, indicating that the observed NK-associated signals arise within a B-cell background rather than from an isolated canonical NK lineage. At the same time, we note that rare composite populations of this kind should be interpreted cautiously. In particular, apparent NK-like B-cell states may in principle be influenced by technical confounders such as doublets or ambient RNA contamination. We therefore regard Supercell 363 as a biologically plausible candidate composite state supported by coordinated RNA/protein evidence and reference context, while acknowledging that further orthogonal validation, such as manual gating or independent reference mapping, would be valuable to establish its identity more definitively.

Together, these examples show that supercell modeling can reveal rare or functionally specialized populations that remain hidden at the conventional cluster level.

### 2.6 t-SNE projections expose rare populations captured by supercells

To illustrate the utility of supercell abstraction, we mapped Supercell 62 and Supercell 363 onto the t-SNE projection of the joint single-cell multi-omics manifold.

**Fig 6:**
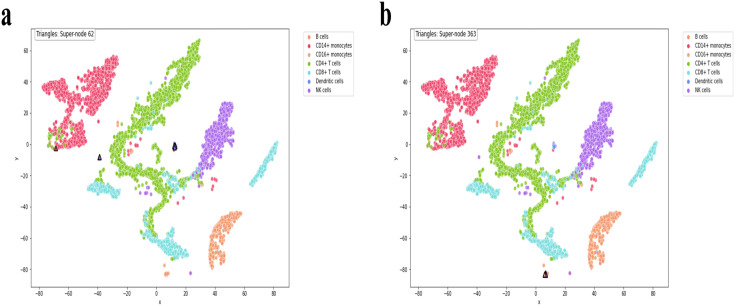
Visualization of supercells in t-SNE embedding. Cells belonging to Supercell 62 and Supercell 363 are highlighted using triangular markers. **(a)** Supercell 62 corresponds to a rare dendritic-cell population. **(b)** Supercell 363 contains a group of B cells with embedded NK cells, representing a rare composite population.

As illustrated in Fig 6a, Supercell 62 (black triangles) aligns almost exclusively with a sparsely distributed dendritic-cell cohort that remains invisible in any single-cell clustering partition. Conventional clustering thus under-samples or fragments this rare population, whereas supercell abstraction consolidates it into an expression-coherent unit, exposing its presence at the mesoscopic scale and demonstrating the utility of supercells for recovering noise-masked minority cell types.

As shown in Fig 6b, Supercell 363 occupies a distinct t-SNE “island” composed chiefly of B cells together with two NK cells. Its clear separation from the main B-cell cluster implies a rare composite population corresponding to the previously described NK-like B cells ([39]). By consolidating these boundary-spanning cells into a single module, the supercell framework remains sensitive to biologically meaningful heterogeneity that would otherwise be lost at the margins of canonical lineages.

In both cases, the supercells occupy spatially isolated regions distinct from the major cellular aggregates, illustrating how supercell modeling can expose biologically meaningful intermediate-scale structure.

### 2.7 scHG achieves state-of-the-art multi-omics clustering performance across six benchmarks

We conducted comprehensive evaluations on six public datasets (see Section 7.5), benchmarking scHG against six state-of-the-art approaches (see Section 7.6) using the **ARI** and **NMI** metrics. Quantitative comparisons are presented in Tables 3 and 4. On the mESC and PBMC_Cao dataset, GSTRPCA failed to produce valid clustering labels because their intermediate representations contained invalid values, which prevented the subsequent label assignment procedure from completing successfully. For the Bmcite dataset, all baseline methods could not be executed to completion due to excessive memory usage during runtime, which exceeded the available MATLAB memory limits under the same experimental environment.

**Table 3:** Performance comparison of different methods in ARI on the considered datasets.

Datasets Methods	PBMC10×	PBMC_Inhouse	Sim	Bmcite	mESC	PBMC_Cao
GBS	0.6750	0.4642	**0.9881**	—	0.6881	0.4231
scAI	0.7208	0.6598	0.9239	—	0.8629	0.4642
SMSC	0.6981	0.6320	**0.9881**	—	0.7329	0.3756
CGD	0.8078	0.6914	0.6901	—	0.6042	0.4512
scMNMF	0.7716	0.5950	0.7278	—	0.8226	0.4153
GSTRPCA	0.5621	0.6295	0.8445	—	—	—
scHG	**0.8774**	**0.7029**	0.9646	**0.6732**	**0.9373**	**0.5299**

**Table 4:** Performance comparison of different methods in NMI on the considered datasets.

Datasets Methods	PBMC10×	PBMC_Inhouse	Sim	Bmcite	mESC	PBMC_Cao
GBS	0.7100	0.4443	**0.9832**	—	0.6440	0.4966
scAI	0.6617	0.7302	0.9425	—	0.8014	0.4749
SMSC	0.6954	0.6930	**0.9832**	—	0.7112	0.5024
CGD	0.7458	0.7989	0.6883	—	0.5873	0.5283
scMNMF	0.7505	0.6468	0.7544	—	0.6564	NaN
GSTRPCA	0.4685	0.6451	0.8614	—	—	—
scHG	**0.8230**	**0.7992**	0.9598	**0.6917**	**0.8589**	**0.5972**

scHG achieves the best performance on most benchmark datasets, including PBMC10×, PBMC_Inhouse, Bmcite, mESC, and PBMC_Cao. On the simulated dataset, although it ranks third in both ARI and NMI, its performance remains highly competitive, with both values exceeding 95%. Overall, the consistent trends in ARI and NMI support the robustness of the proposed framework across diverse datasets.

**Fig 7:**
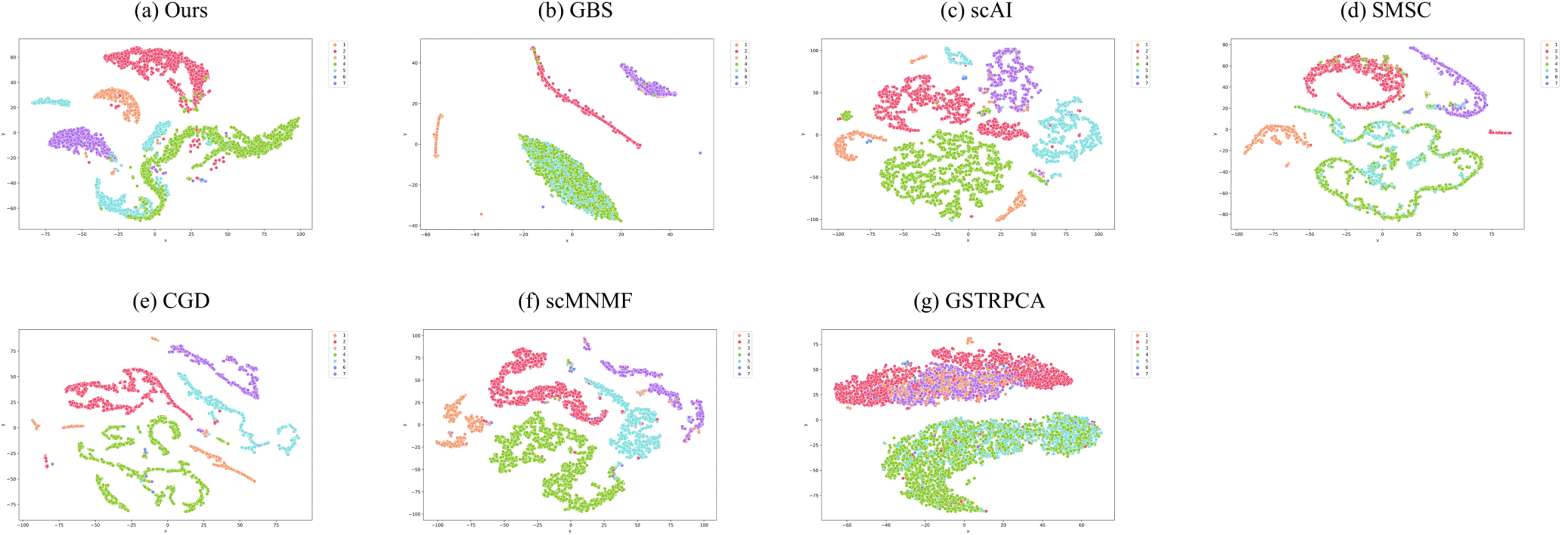
Comparative t-SNE Visualization of Latent Representations on PBMC10 × Dataset: (a) scHG, (b) GBS, (c) scAI, (d) SMSC, (e) CGD, (f) scMNMF, (g) GSTRPCA.

To evaluate embedding quality, we project high-dimensional omics data from all six benchmark datasets into two-dimensional space using t-SNE. Fig 7 presents a representative visualization, while the results for the remaining datasets are provided in Figs A-D in S1 Appendix. SMSC generated only one-dimensional embeddings on mESC datasets, which are unsuitable for t-SNE.

Taking PBMC10× as an example, scHG produces compact and well-separated clusters, whereas several baselines show either higher intra-cluster dispersion or stronger inter-cluster overlap. Similar patterns are observed across the remaining datasets, indicating that supercell construction and pruning improve both intra-cluster compactness and inter-cluster separability.

### 2.8 High-order supercell strategy achieves state-leading runtime efficiency on large-scale multi-omics datasets

**Fig 8:**
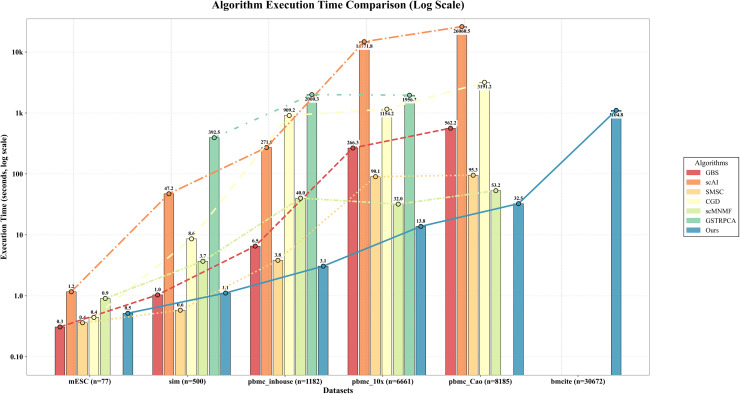
Execution time comparison (Log Scale) of different methods on the considered datasets.

We benchmarked computational efficiency across six comparative methods and our approach on public datasets (Table 5 & Fig 8).

**Table 5:** Performance comparison of different methods in time(s) on the considered datasets.

Datasets Methods	PBMC10×	PBMC_Inhouse	Sim	Bmcite	mESC	PBMC_Cao
GBS	266.324	6.546	1.036	—	**0.309**	562.208
scAI	14771.801	271.596	47.234	—	1.167	26060.514
SMSC	90.112	3.823	**0.581**	—	0.362	95.281
CGD	1154.190	909.180	8.617	—	0.442	3191.165
scMNMF	31.957	40.033	3.691	—	0.905	53.151
GSTRPCA	1956.157	2000.284	392.497	—	—	—
scHG	**13.781**	**3.064**	1.112	**1104.834**	0.518	**32.507**

On smaller datasets (mESC and Sim), scHG shows competitive but not leading runtime. In contrast, on larger datasets including PBMC_Inhouse, PBMC10×, PBMC_Cao, and Bmcite, it is the fastest method among those successfully executed. This trend is consistent with the reduction in effective problem size introduced by supercell construction, which becomes increasingly advantageous as dataset size grows.

### 2.9 Memory usage comparison across methods

To further evaluate the computational scalability of scHG, we additionally compared the peak memory consumption of scHG and baseline methods across datasets of different sizes. The peak memory usage was recorded under the same MATLAB environment for all methods.

**Fig 9:**
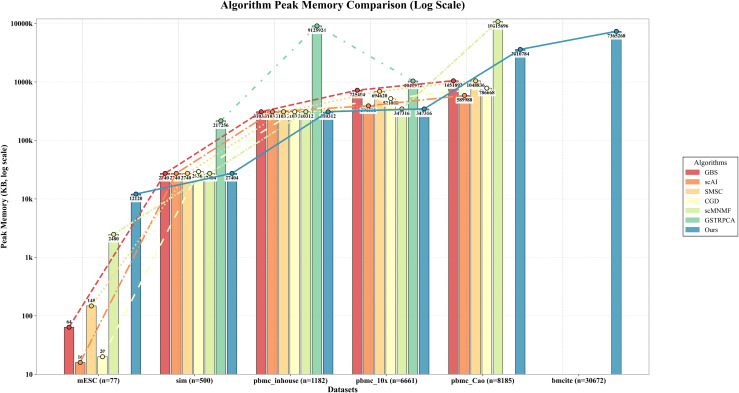
Peak memory consumption comparison (Log Scale) of different methods on the considered datasets.

As shown in Fig 9, the peak memory consumption of scHG remains comparable to that of existing methods across datasets. Although some baseline methods require slightly lower peak memory on certain datasets, several methods fail to complete execution due to excessive memory consumption on large datasets. In contrast, scHG successfully completes execution while maintaining moderate memory usage.

These results indicate that the proposed framework achieves a favorable balance between runtime efficiency and memory consumption, enabling practical application to large-scale multi-omics datasets.

### 2.10 High-order refinement enhances supercell purity by correcting second-order misclassifications

To illustrate the benefit of incorporating high-order information, we analysed the PBMC10× dataset (6661 cells, S5 Table). Using only second-order co-occurrence, the fused cross-omic graph partitions the data into 4802 supercells. Among these, seven supercells (IDs 62, 63, 363, 453, 1271, 1852, 1917) contain at least three cells and span two or more ground-truth labels. After augmenting the graph with higher-order neighborhoods and degree-centrality pruning, many misassigned cells are removed.

On the PBMC10× dataset, the pruning step removes 11 out of 6,661 cells (0.17%). Although the removal ratio is extremely small, the pruned cells predominantly correspond to low-degree, peripheral nodes in the supercell graph. Eliminating these low-confidence cells helps correct erroneous second-order co-occurrences and improves the purity of the resulting supercells.

We quantify this effect with ${\text{Recall}}_{sc}$:


$$\begin{array}{c} \begin{array}{c} {Recall}_{sc}\;=\;\frac{1}{S}\sum_{s=1}^{S}\frac{{TP}_{s}}{{TP}_{s}+{FN}_{s}}, \end{array} \end{array}$$ (3)

where TP_*s*_ and FN_*s*_ counts, respectively, denote the mislabelled cells successfully pruned and those that remain in supercell *s*. The confusion matrix in Table 6 yields a ${\text{Recall}}_{sc}$ of 75.97%, indicating that three-quarters of the second-order misclassifications are corrected by the high-order refinement.

**Table 6:** Cell–level confusion statistics for the seven supercells (IDs 62, 63, 363, 453, 1771, 1852, 1917) in the PBMC10 × dataset before and after high-order refinement.

	62	63	363	453	1771	1852	1917
TN	19	17	10	4	1	2	1
FN	2	1	2	0	0	0	0
FP	16	9	21	5	1	4	1
TP	9	1	0	3	1	1	1

**Fig 10:**
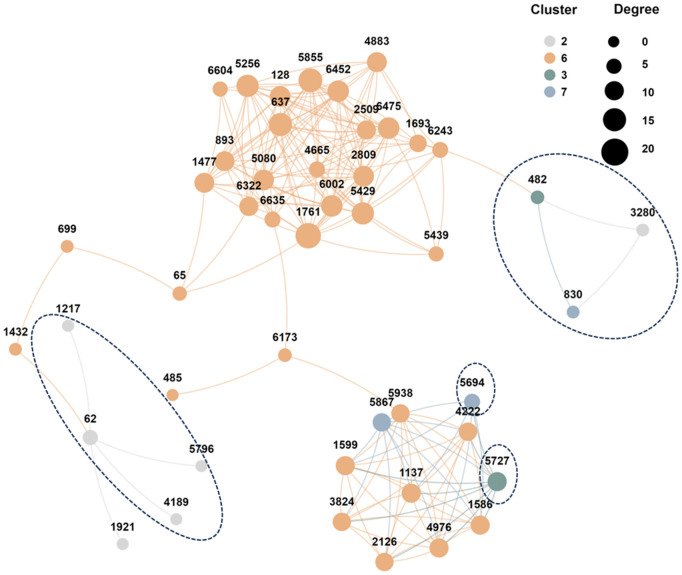
Connectivity graph of Supercell 62 before high-order pruning. Node size indicates degree centrality and colors denote ground-truth clusters. Dashed ellipses mark low-centrality cells removed during high-order refinement. (This plot was generated using the CNSknowall platform (https://cnsknowall.com), a comprehensive web service for data analysis and visualization.).

Fig 10 visualizes Supercell 62. Of eleven initially mis-grouped cells, nine are removed by the high-order refinement, and these cells show lower degree centrality than retained members. This example illustrates how high-order pruning improves supercell purity.

### 2.11 Comparison with additional baselines

To better position scHG within the current landscape of single-cell multi-omics integration, we further compared it against several strong and widely used baselines, including Seurat WNN, Harmony followed by clustering, MOFA + , and MultiVI. These methods represent complementary methodological paradigms, including weighted nearest-neighbor integration, batch-corrected embedding, latent factor modeling, and deep generative learning. We included them as additional references beyond the baselines considered in our main benchmark.

Tables 7 and 8 summarize the ARI and NMI results, respectively. Overall, the newly added baselines achieve competitive performance on several datasets, confirming that they constitute a stronger comparison set than classical methods alone. Rather than uniformly dominating all datasets, scHG shows the most robust overall performance and remains among the top-performing methods across benchmarks. In particular, it achieves the best results on the more heterogeneous or biologically challenging datasets, including Bmcite, mESC, and PBMC_Cao, which is consistent with our design motivation of constructing topology-aware, cross-omics-consistent supercells for scalable yet rare-population-sensitive clustering.

**Table 7:** Performance comparison of additional baselines and scHG in ARI on the benchmark datasets.

Datasets Methods	PBMC10×	PBMC_Inhouse	Sim	Bmcite	mESC	PBMC_Cao
MOFA+	0.7428	0.6187	0.9185	0.5214	0.8016	0.4382
Seurat WNN	**0.8826**	0.6812	0.9684	0.6189	0.8894	0.4876
Harmony+clustering	0.7814	0.6538	0.9327	0.6364	0.8463	0.5038
MultiVI	0.8698	**0.7113**	0.9413	0.5637	0.9071	0.4591
scVI	0.7987	0.6649	**0.9721**	0.5812	0.8625	0.4715
**scHG**	0.8774	0.7029	0.9646	**0.6732**	**0.9373**	**0.5299**

**Table 8:** Performance comparison of additional baselines and scHG in NMI on the benchmark datasets.

Datasets Methods	PBMC10×	PBMC_Inhouse	Sim	Bmcite	mESC	PBMC_Cao
MOFA+	0.7216	0.7014	0.9248	0.5569	0.7648	0.5112
Seurat WNN	**0.8314**	0.7763	0.9638	0.6475	0.8297	0.5679
Harmony+clustering	0.7534	0.7446	0.9362	0.6713	0.7881	0.5827
MultiVI	0.8178	**0.8069**	0.9454	0.5984	0.8419	0.5396
scVI	0.7695	0.7581	**0.9713**	0.6198	0.8065	0.5518
**scHG**	0.8230	0.7992	0.9598	**0.6917**	**0.8589**	**0.5972**

Among the newly added methods, Seurat WNN performs best on PBMC10×, MultiVI achieves the strongest performance on PBMC_Inhouse, and scVI performs best on the simulated dataset. These results suggest that neighborhood-based or deep generative integration strategies can be particularly effective when the data structure is relatively regular or cluster boundaries are more clearly defined. Harmony+clustering and MOFA+ are also competitive on some datasets, but their performance is less consistent across benchmarks. By comparison, scHG provides a more favorable balance between robustness, clustering accuracy, and sensitivity to minority populations, further supporting its practical utility as a general multi-omics clustering framework.

Where necessary, we used the modality-compatible implementation or the closest standard configuration of each baseline for the corresponding dataset, following common practice in the literature. These additional baselines cover neighborhood-based integration (Seurat WNN), linear latent factor modeling (MOFA+), batch-corrected embedding (Harmony), and deep generative modeling (MultiVI, scVI), thereby providing a broader and more up-to-date comparison set.

## 3 Discussion

### 3.1 Ablation analysis

To validate the effectiveness of the proposed angle-aware metric and probabilistic pruning modules, we performed detailed ablation studies across all six datasets (referenced in Section 7.5). Our experimental protocol establishes a baseline model employing Euclidean distance without probabilistic pruning, maintaining identical hyperparameters and solution methodologies to ensure comparative fairness.

**Fig 11:**
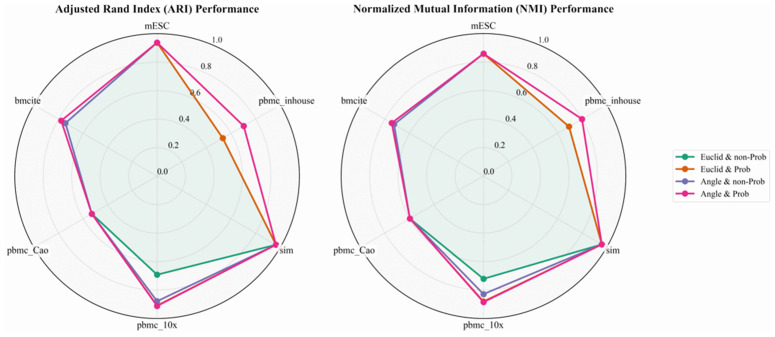
Ablation experiment results (NMI & ARI) on the considered datasets.

Quantitative performance improvements are evaluated across eight metrics in Table 9: Accuracy (**ACC**; [40]), Normalized Mutual Information (**NMI**; [41,42]), **Purity** ([43]), **F1-score** ([44]), **Precision** ([44]), **Recall** ([44]), Rand Index (**RI**; [45]), and Adjusted Rand Index (**ARI**; [46]). In particular, we present the comparison of **ARI** and **NMI** through Fig 11.

**Table 9:** Ablation results of angle-aware metric and probabilistic pruning modules in six datasets across eight clustering evaluation metrics; E stands for Euclidean metric and A for angle-aware metric.

Metric	Module	Dataset					
PBMC10×	PBMC_Inhouse	Sim	Bmcite	mESC	PBMC_Cao
ACC	E	0.7607	0.6387	**0.9880**	0.7490	**0.9870**	**0.6316**
	E+Prune	0.9548	0.6387	**0.9880**	**0.7539**	**0.9870**	**0.6316**
	A	0.9023	**0.8029**	**0.9880**	0.7490	**0.9870**	**0.6316**
	A+Prune	**0.9553**	**0.8029**	**0.9880**	**0.7539**	**0.9870**	**0.6316**
NMI	E	0.7215	0.6939	**0.9598**	0.7257	**0.8589**	**0.5972**
	E+Prune	0.8807	0.6939	**0.9598**	**0.7444**	**0.8589**	**0.5972**
	A	0.8286	**0.7992**	**0.9598**	0.7257	**0.8589**	**0.5972**
	A+Prune	**0.8847**	**0.7992**	**0.9598**	**0.7444**	**0.8589**	**0.5972**
Purity	E	0.9541	0.9332	**0.9880**	0.7498	**0.9870**	**0.6318**
	E+Prune	0.9602	0.9332	**0.9880**	**0.7549**	**0.9870**	**0.6318**
	A	0.9580	**0.9915**	**0.9880**	0.7498	**0.9870**	**0.6318**
	A+Prune	**0.9632**	**0.9915**	**0.9880**	**0.7549**	**0.9870**	**0.6318**
F1-score	E	0.7544	0.6408	**0.9744**	0.7747	**0.9816**	**0.6446**
	E+Prune	0.9313	0.6408	**0.9744**	**0.8037**	**0.9816**	**0.6446**
	A	0.9069	**0.7758**	**0.9744**	0.7747	**0.9816**	**0.6446**
	A+Prune	**0.9334**	**0.7758**	**0.9744**	**0.8037**	**0.9816**	**0.6446**
Precision	E	0.9141	0.8633	**0.9824**	0.6889	**0.9936**	**0.4788**
	E+Prune	0.9426	0.8633	**0.9824**	**0.7398**	**0.9936**	**0.4788**
	A	0.9451	**0.9849**	**0.9824**	0.6889	**0.9936**	**0.4788**
	A+Prune	**0.9463**	**0.9849**	**0.9824**	**0.7398**	**0.9936**	**0.4788**
Recall	E	0.6422	0.5095	**0.9665**	**0.8849**	**0.9699**	**0.9861**
	E+Prune	0.9204	0.5095	**0.9665**	0.8797	**0.9699**	**0.9861**
	A	0.8716	**0.6399**	**0.9665**	**0.8849**	**0.9699**	**0.9861**
	A+Prune	**0.9208**	**0.6399**	**0.9665**	0.8797	**0.9699**	**0.9861**
RI	E	0.8977	0.8222	**0.9859**	0.9448	**0.9740**	**0.8029**
	E+Prune	0.9668	0.8222	**0.9859**	**0.9539**	**0.9740**	**0.8029**
	A	0.9562	**0.8848**	**0.9859**	0.9448	**0.9740**	**0.8029**
	A+Prune	**0.9679**	**0.8848**	**0.9859**	**0.9539**	**0.9740**	**0.8029**
ARI	E	0.6923	0.5329	**0.9646**	0.7438	**0.9373**	**0.5299**
	E+Prune	0.9095	0.5329	**0.9646**	**0.7778**	**0.9373**	**0.5299**
	A	0.8783	**0.7029**	**0.9646**	0.7438	**0.9373**	**0.5299**
	A+Prune	**0.9122**	**0.7029**	**0.9646**	**0.7778**	**0.9373**	**0.5299**

For PBMC10×, PBMC_Inhouse, and Bmcite datasets, models incorporating angle-aware metrics and probabilistic pruning demonstrated substantial performance gains relative to Euclidean-distance counterparts without pruning: **NMI** improvements of 16.32%, 10.53%, and 1.87%; and **ARI** improvements of 21.99%, 17.00%, and 3.40% respectively. On the remaining three datasets, these components maintained performance without degradation. Collectively, our ablation results confirm that each proposed component contributes non-negligibly to performance enhancement, supporting the overall efficacy of scHG design.

### 3.2 Performance on different cluster numbers

**Fig 12:**
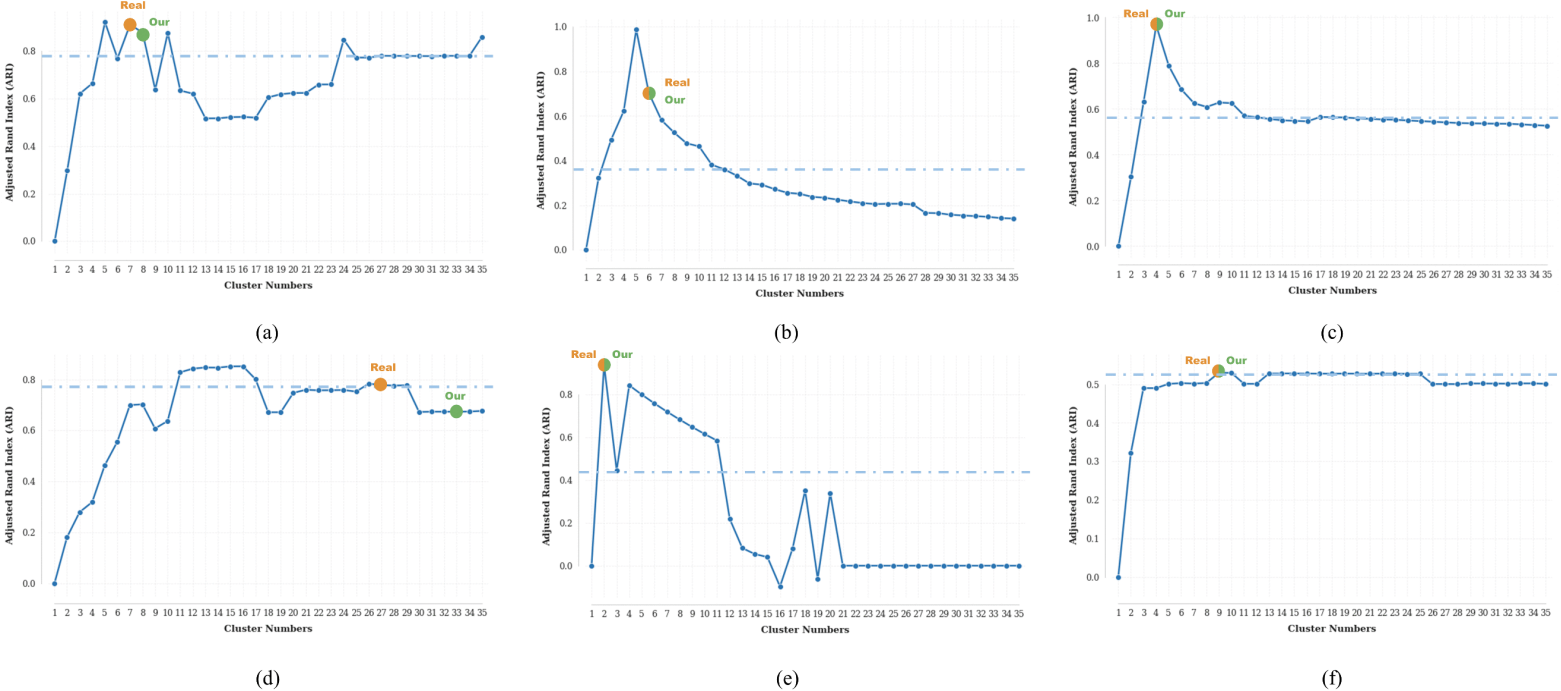
ARI variation across six benchmark datasets: **(a)** PBMC10 × , **(b)** PBMC_Inhouse, **(c)** Sim, **(d)** Bmcite, (e) mESC, **(f)** PBMC_Cao.Dots indicate: yellow (ground-truth cluster numbers), green (algorithm-derived optimal cluster numbers). Dashed indicate: blue (top-10 ARI threshold).‌‌.

**Fig 13:**
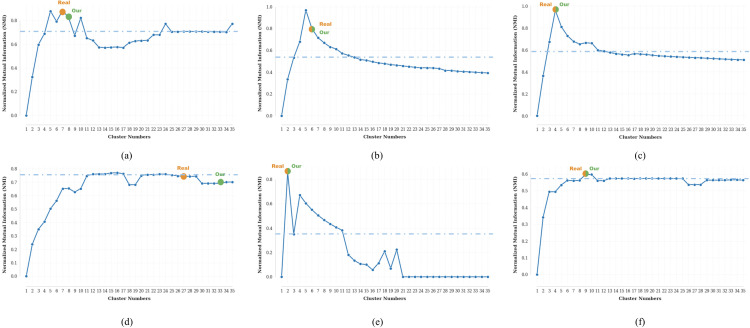
NMI variation across six benchmark datasets: (a) PBMC10 × , (b) PBMC_Inhouse, (c) Sim, (d) Bmcite, (e) mESC, (f) PBMC_Cao. Dots indicate: yellow (ground-truth cluster numbers), green (algorithm-derived optimal cluster numbers). Dashed indicate: blue (top-10 NMI threshold).

We conducted systematic evaluations of clustering sensitivity across benchmark datasets by measuring **ARI**/**NMI** variation with cluster number (Figs 12 and 13). On four datasets (PBMC_Inhouse, Sim, mESC, PBMC_Cao), scHG achieves exact alignment between algorithm-optimized (green) and ground-truth (yellow) cluster numbers, with identical **ARI**/**NMI** rankings. For the remaining two datasets (PBMC10×, Bmcite), while minor discrepancies existed in cluster number estimation, our model achieved indistinguishable performance compared to ground-truth configurations.

Across half of the benchmark datasets (PBMC10×, Bmcite, PBMC_Cao), the top-10 thresholds achieve 84.6–99.6% of the maximum **ARI** and 80.0–98.3% of the maximum **NMI**, indicating high robustness to cluster number selection.

For the remaining datasets (PBMC_Inhouse, Sim, mESC), the top-10 thresholds achieve 36.6–58.5% of the maximum **ARI** and 40.6–61.2% of the maximum **NMI**, while maintaining 100% accuracy in cluster number estimation (Figs 12 and 13), underscoring the method’s capability to precisely determine cluster numbers across sensitivity ranges.

In all experiments, scHG estimated the number of clusters adaptively using the modality-weighted fusion strategy in Eq. (31), without using ground-truth labels. For the competing methods, when the number of clusters was required as an input parameter, we provided the true number of cell types/classes in the corresponding dataset. We include this setting here to ensure transparency in the benchmarking protocol.

### 3.3 Robustness analysis of hyperparameters α and β

**Fig 14:**
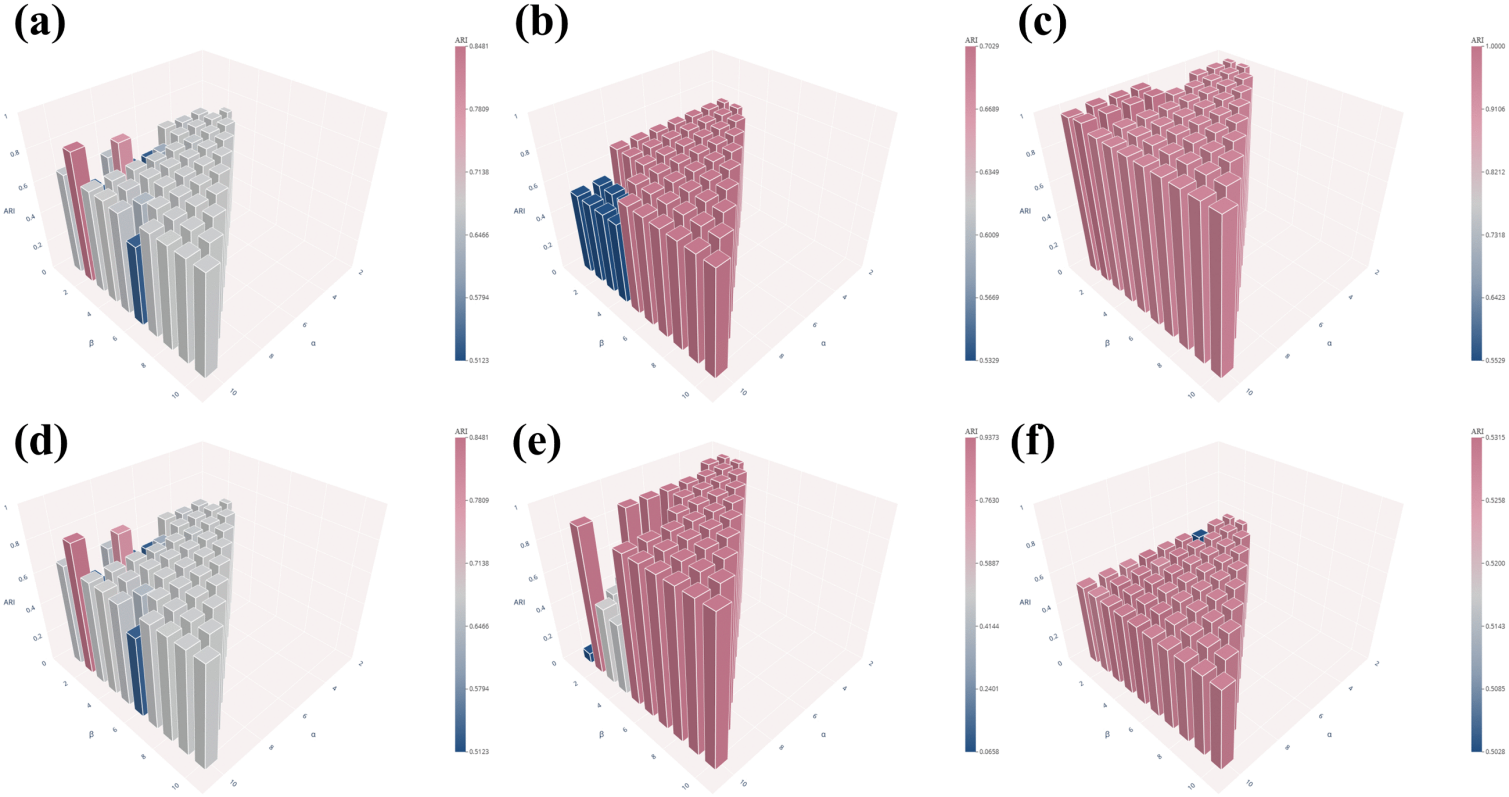
Clustering performance (ARI) across the (*α*, *β*) hyperparameter space on six datasets: (a) PBMC10 × , (b) PBMC_Inhouse, (c) Sim, (d) Bmcite, (e) mESC, (f) PBMC_Cao.

**Fig 15:**
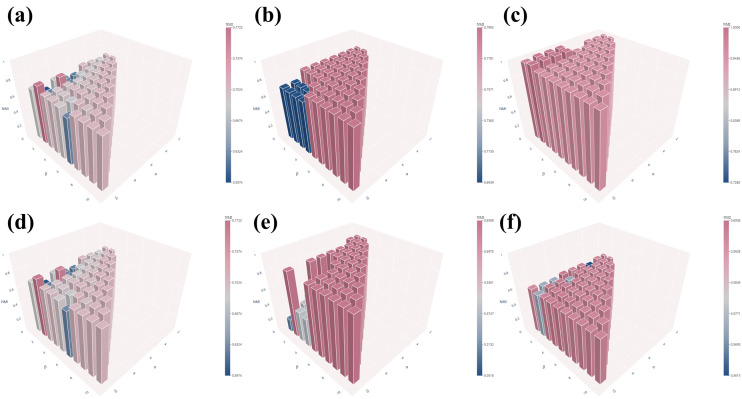
Clustering performance (NMI) across the (*α*, *β*) hyperparameter space on six datasets: (a) PBMC10 × , (b) PBMC_Inhouse, (c) Sim, (d) Bmcite, (e) mESC, (f) PBMC_Cao.

During supercell construction, two hyperparameters, α and β, are introduced to regulate the aggregation process. To systematically examine their impact on clustering performance, we performed a grid-based hyperparameter analysis in which α was varied from 1 to 10 and β was varied from 1 to α. The clustering performance obtained under different (α,β) configurations is visualized as a three-dimensional bar plot in Figs 14 and 15, facilitating a comprehensive evaluation of performance trends and parameter sensitivity across the explored hyperparameter space.

As shown in Figs 14 and 15, clustering performance remains largely stable across the explored (α,β) hyperparameter space on all six datasets, with minor deviations observed only under a small number of imbalanced parameter settings. This observation indicates that scHG is not overly sensitive to precise hyperparameter tuning and exhibits strong robustness across a wide range of parameter settings, thereby reducing the risk of performance degradation in practical applications.

### 3.4 Sensitivity analysis of similarity threshold γ

**Fig 16:**
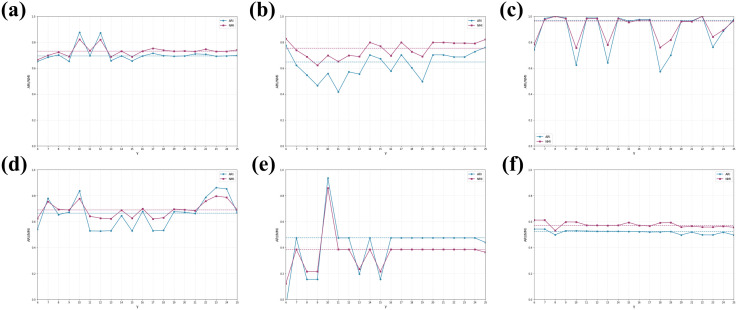
Clustering performance (ARI & NMI) across the *γ* hyperparameter space on six datasets: (a) PBMC10 × , (b) PBMC_Inhouse, (c) Sim, (d) Bmcite, (e) mESC, (f) PBMC_Cao.Solid lines denote clustering performance under different values of *γ*, while dashed lines indicate the median ARI (blue) or NMI (red) for each dataset.

During the construction of the cell similarity matrix **A**(*v*), the neighborhood of each cell was first identified by extracting the top (γ+1) most correlated cells (excluding self) from matrix **PCC**(*v*), based on which **A**(*v*) was subsequently computed. To further examine the effect of the neighborhood size parameter γ on clustering performance, we systematically varied γ from 6 to 25 (resulting in 20 distinct settings). Clustering performance was evaluated using ARI and NMI for each configuration, and the corresponding results are presented as line plots in Fig 16, enabling a clear assessment of performance trends and parameter sensitivity.

Fig 16 illustrates the effect of the neighborhood size parameter γ on clustering performance across six datasets. On the PBMC10×, PBMC_Inhouse, Bmcite and PBMC_Cao datasets, both ARI and NMI vary smoothly as γ changes, indicating that clustering performance is largely insensitive to moderate variations in neighborhood size. In contrast, more evident performance fluctuations are observed on the Sim, and mESC datasets, suggesting a higher sensitivity to the choice of γ in these cases.

Across all datasets, a consistent empirical tendency can be observed: values of γ associated with relatively stronger clustering performance are closely related to the dimensionality of the omics features used for clustering. Specifically, when the maximum number of features in the employed omics dataset is below 5000, setting γ=10 generally yields more favorable clustering results, whereas γ=20 tends to perform better for datasets with higher feature dimensionality. This empirical tendency motivates the choice of γ in scHG configuration, as described in Section 7.7.

### 3.5 Randomness analysis of supercell construction

During supercell construction, random pruning is introduced. Unless otherwise stated, all results reported in Section 4 were obtained using a fixed random seed, ensuring full reproducibility. To explicitly assess the impact of the introduced randomness, we performed 20 independent clustering runs on each of the six datasets, and summarize the resulting performance statistics in Table 10.

**Table 10:** Randomness Analysis of scHG Across Six Datasets (Mean ± Variance over 20 Runs).

Datasets Metrics	PBMC10×	PBMC_Inhouse	Sim	Bmcite	mESC	PBMC_Cao
**ARI**	**0.8774**±0.0000	**0.7029**±0.0000	**0.9646**±0.0000	**0.6732**±0.0000	**0.9373**±0.0000	**0.5299**±0.0000
**NMI**	**0.8230**±0.0000	**0.7992**±0.0000	**0.9598**±0.0000	**0.6917**±0.0000	**0.8589**±0.0000	**0.5972**±0.0000

For both clustering metrics (ARI and NMI), the observed variance is consistently zero across all datasets, indicating that scHG yields identical clustering outcomes across repeated runs. This result suggests that the randomness introduced during supercell construction does not propagate to the final clustering results and exhibits strong robustness to stochastic operations.

### 3.6 Convergence behavior of the omics-weighted optimization

**Fig 17:**
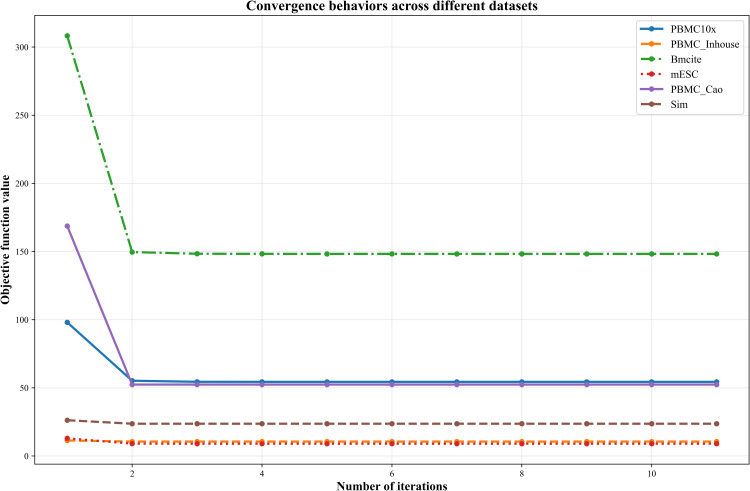
Convergence behavior of the omics-weighted optimizer across datasets. Objective value as a function of the iteration number for six representative datasets (PBMC10 × , PBMC_inhouse, Bmcite, mESC, PBMC_Cao, and Sim). Each curve corresponds to one dataset.‌‌.

To further examine the optimization dynamics of the proposed framework, we analyzed the convergence behavior of the objective function across six datasets. Specifically, we recorded the objective value at each iteration during optimization and summarized the results in Fig 17.

As shown in Fig 17, the objective value decreases sharply during the first few iterations for all datasets, followed by rapid stabilization. In most cases, the objective function reaches a near-stationary state within two to three iterations, after which only negligible changes are observed. This behavior is consistent across datasets with varying sizes and characteristics. The monotonic decrease of the objective value confirms the stability of the alternating optimization procedure, while the rapid plateau indicates that the proposed optimizer efficiently reaches a stable solution with very few iterations. These observations demonstrate that the omics-weighted optimization exhibits stable and efficient convergence behavior in practice, supporting its applicability to diverse multi-omics datasets.

### 3.7 Scalability on large-scale datasets

To further evaluate the scalability of scHG, we conducted an additional experiment on a simulated dataset containing 100,000 cells. The dataset includes two omics modalities (ATAC and RNA) with 5,000 and 2,000 features respectively, and 15 ground-truth clusters.

The results show that scHG successfully processes this large-scale dataset and achieves high clustering performance (e.g., ARI = 0.9967, NMI = 0.9927), with the objective function converging rapidly within a few iterations. In contrast, baseline methods could not be executed on this dataset due to excessive memory requirements exceeding the available system memory. Together, these results demonstrate the ability of scHG to scale to datasets with 100,000 cells while maintaining stable optimization behavior.

### 3.8 Limitations

Although scHG substantially improves computational efficiency by compressing cell-level data into supercells and performing downstream learning on a reduced graph, the current implementation may still face practical limitations on atlas-scale datasets containing hundreds of thousands to millions of cells. In particular, several stages of the pipeline, including neighborhood graph construction, second-order co-occurrence computation, and sparse matrix operations during graph fusion and clustering, may become increasingly demanding as dataset size grows. From an implementation perspective, however, many of these components are naturally amenable to further acceleration through GPU computing and parallelization, such as k-nearest-neighbor search, sparse graph construction, matrix fusion, and graph-based message passing. In future work, distributed or mini-batch implementations may provide an additional route to extending scHG to even larger multi-omics atlases.

## 4 Conclusion

We present scHG, a high-order supercell framework that achieves both scalability and subtype-aware resolution in multi-omics integration. By abstracting expression-coherent cells into supercells, scHG applies an angle-aware metric and a modality-weighted block coordinate descent optimization to cluster supercells efficiently.

scHG processes 30000 cells in under 20 minutes, consistently delivering state-of-the-art ARI and NMI across five real-world datasets. Without relying on prior cluster labels, it retrieves clinically relevant B-cell targets and resolves heterogeneous subpopulations within reference-defined T-cell clusters. The supercell perspective further exposes rare dendritic-cell clusters and NK-like B-cell intermediates that conventional pipelines fail to detect. Ablation studies demonstrate that the high-order graph model—integrating second-order co-occurrence neighbors, angle-aware metrics, and stochastic pruning—boosts performance by an average of 14% across eight clustering metrics relative to second-order graph baselines, while enabling adaptive determination of cluster numbers without prior assumptions.

Together, these findings underscore the power of the supercell paradigm, grounded in high-order graph representations, to uncover rare immune states and decode tissue complexity. Its inherently graph-based architecture provides a natural avenue for incorporating spatial context and additional omics layers, offering a forward-looking analytical framework with strong potential for deeper biological insight and clinically actionable discovery.

## 5 Materials and methods

Consider a collection of *V* multi-omics datasets $\{𝐗\left(v\right){\}}_{v=1}^{V}$, where each matrix $𝐗\left(v\right)\in {\mathbb{R}}^{n\times m_{v}}$ represents a distinct omics data. Here *n* denotes the number of cells, and *m*_*v*_ the number of features for the *v*-th omics data **X**(*v*). Let ${𝐱}^{i}\left(v\right)$ denote the *i*-th row vector (*i*-th cell) and ${𝐱}_{j}\left(v\right)$ the *j*-th column vector (*j*-th feature) of **X**(*v*), corresponding to biological features such as gene expression (RNA-seq), surface protein abundance (antibody-derived tags (ADT)), or chromatin accessibility (ATAC-seq).

### 5.1 Angle-aware metric

In traditional omics clustering analyses, similarity between cells is frequently quantified using Euclidean distance, whereby smaller distances indicate greater similarity. In contrast, Eq. (4) defines an alternative angle-aware metric: the Pearson correlation coefficient (PCC) between cells.


$$\begin{array}{c} \begin{array}{c} PCC\left({𝐱}^{i}\left(v\right),{𝐱}^{j}\left(v\right)\right)=\frac{{\left({𝐱}^{i}\left(v\right)-{\bar{𝐱}}^{i}\left(v\right)\right)}^{T}\left({𝐱}^{j}\left(v\right)-{\bar{𝐱}}^{j}\left(v\right)\right)}{\|{𝐱}^{i}\left(v\right)-{\bar{𝐱}}^{i}\left(v\right){\|}_{2}\|{𝐱}^{j}\left(v\right)-{\bar{𝐱}}^{j}\left(v\right){\|}_{2}}, \end{array} \end{array}$$ (4)

Where ${𝐱}^{i}\left(v\right)$ and ${𝐱}^{j}\left(v\right)$ are any two cells in the *v*-th omics, respectively. Notably, the Pearson correlation coefficient can be interpreted as the cosine of the angle between mean-centered feature vectors, which makes the similarity invariant to scale differences across cells.

**Fig 18:**
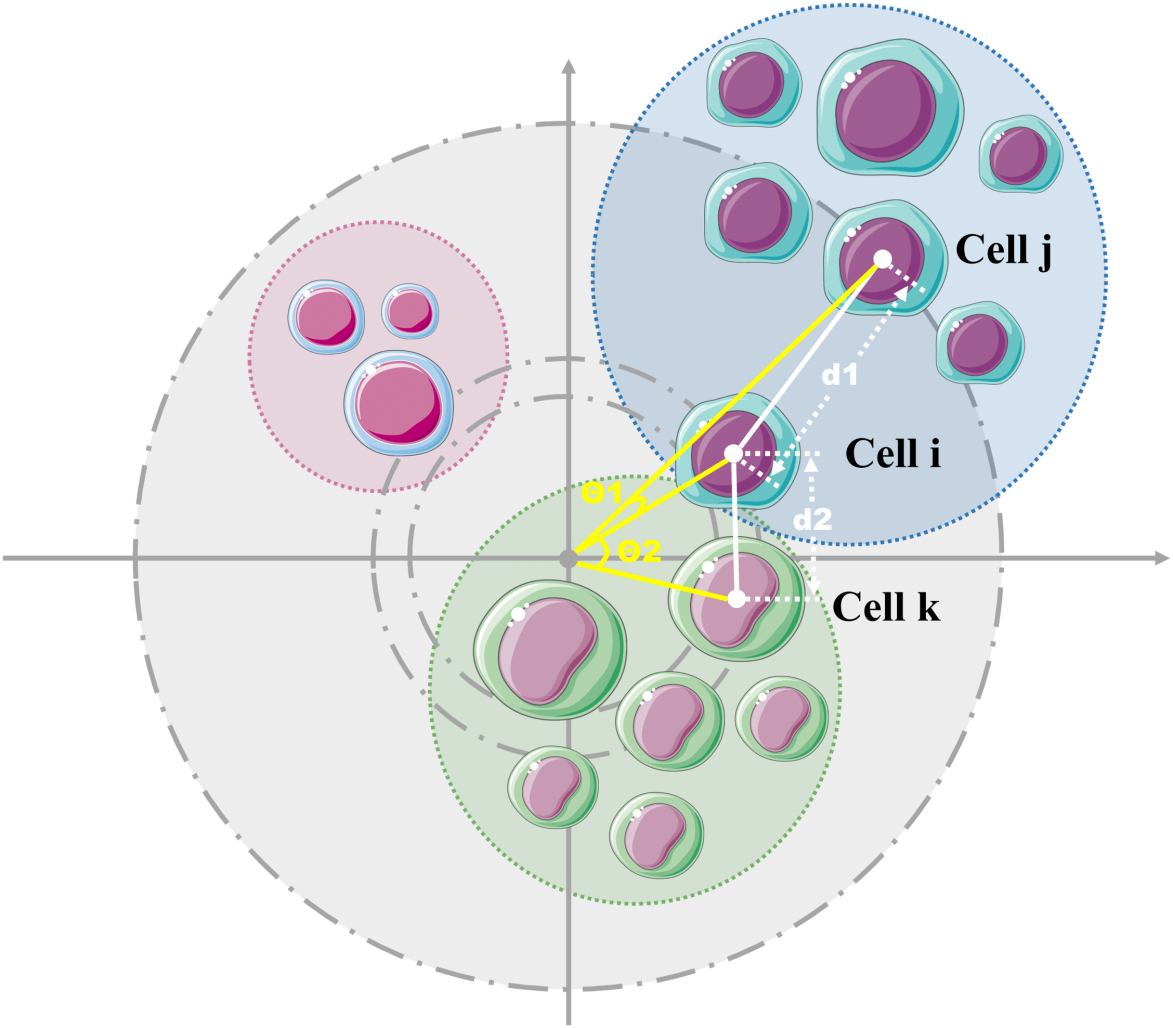
Comparison between Euclidean and angle-aware metrics. Although Euclidean distance suggests grouping cells *i* and *k*, the angular metric based on Pearson correlation indicates higher similarity between cells *i* and *j*, illustrating the advantage of the angle-aware metric.

Compared with standard cosine similarity, PCC first removes the mean of each cell vector before measuring the angular similarity, thereby emphasizing relative variation patterns rather than absolute expression magnitudes. Building on the angle-aware metric introduced earlier, we can distinguish cell pairs that are geographically proximate (small Euclidean distance) but lack strong linear correlation. As illustrated in Fig 18, *d*_1_ > *d*_2_ indicates greater Euclidean separation between cells *i* and *j* than between *i* and *k*, implying lower Euclidean-based similarity. Consequently, these cells (*i* and *k*) would typically be grouped together under traditional clustering. However, the angular metric reveals that $\theta_{1}<\theta_{2}$, leading to $PCC\left({𝐱}^{i},{𝐱}^{j}\right)>PCC\left({𝐱}^{i},{𝐱}^{k}\right)$. This demonstrates significantly higher linear-correlation similarity between cells *i* and *j* than between *i* and *k*, which is more reasonable.

Given these comparative advantages of the angle-aware metric over Euclidean distance, our algorithm employs angle-aware metric as the primary similarity measure. The effectiveness of this design choice is further validated through ablation experiments (Section 5.1), where replacing the angle-aware metric with Euclidean distance leads to consistent performance degradation across several datasets.

### 5.2 High order neighbor-aware coarsening graph clustering framework

The cell similarity matrix $𝐀(v)=(a_{ij}{)}_{n\times n}$ for the *v*-th omic is constructed using the γ-proximity linear similarity metric. Specifically, we first compute the Pearson correlation matrix **PCC**(*v*) of matrix **X**(*v*) by:


$$\begin{array}{c} \begin{array}{c} PCC(v{)}_{ij}=PCC\left({𝐱}^{i}\left(v\right),{𝐱}^{j}\left(v\right)\right) \end{array} \end{array}$$ (5)

Then for each cell *i*, we extract its γ+1 nearest neighbors (excluding self) from **PCC**(*v*) to form the truncated ordered distance matrix $\tilde{PCC}\left(v\right)$, yielding the similarity coefficients:


$$\begin{array}{c} \begin{array}{c} a_{ij}(v)=\left\{ \begin{array}{cc} \frac{\tilde{PCC}(v{)}_{i(\gamma +1)}-\tilde{PCC}(v{)}_{ij}}{\gamma \cdot \tilde{PCC}(v{)}_{i(\gamma +1)}-\sum_{j=1}^{\gamma }\tilde{{PCC}^{\prime }}(v{)}_{ij}}, & \text{If }1\leq j\leq \gamma ,\\ 0, & \text{else.} \end{array}\right. \end{array} \end{array}$$ (6)

Given *C* cell clusters, let ${𝒞}_{p}$ denote the *p*-th cluster. The inter-cluster similarity between ${𝒞}_{p}$ and its complement $\overset{―}{{𝒞}_{p}}$ is defined as:


$$\begin{array}{c} \begin{array}{c} sim({𝒞}_{p},\bar{{𝒞}_{p}})=\sum_{i\in {𝒞}_{p}}\sum_{j\in \bar{{𝒞}_{p}}}(a_{ij}(v)+a_{ji}(v)). \end{array} \end{array}$$ (7)

The total inter-cluster similarities across all clusters and their complements is quantified as:


$$\begin{array}{c} \begin{array}{c} \sum_{p=1}^{C}sim({𝒞}_{p},\bar{{𝒞}_{p}})=\sum_{p=1}^{C}\sum_{i\in {𝒞}_{p}}\sum_{j\in \bar{{𝒞}_{p}}}(a_{ij}(v)+a_{ji}(v)). \end{array} \end{array}$$ (8)

To account for omics-specific contributions, we introduce modality weights $\{\omega \left(v\right){\}}_{v=1}^{V}$, yielding the weighted multi-omics similarity:


$$\begin{array}{c} \begin{array}{c} \sum_{v=1}^{V}\omega \left(v\right)\sum_{p=1}^{C}\sum_{i\in {𝒞}_{p}}\sum_{j\in \bar{{𝒞}_{p}}}(a_{ij}(v)+a_{ji}(v)). \end{array} \end{array}$$ (9)

To seek maximally separable partitions, the total multi-omics similarity should be minimized through the following constrained optimization:


$$\begin{array}{c} \begin{array}{c} \min_{\{{𝒞}_{p}{\}}_{p=1}^{C},\{\omega \left(v\right){\}}_{v=1}^{V}}\sum_{v=1}^{V}\omega (v)\sum_{p=1}^{C}\sum_{i\in {𝒞}_{p}}\sum_{j\in \bar{{𝒞}_{p}}}(a_{ij}(v)+a_{ji}(v)),\;s.t.{⋃}_{p=1}^{C}{𝒞}_{p}=𝒞,\forall p\neq q\in {\mathbb{Z}}_{\left[1,C\right]},{𝒞}_{p}⋂{𝒞}_{q}=\emptyset ,\;\forall v\in {\mathbb{Z}}_{\left[1,V\right]},\sum_{v=1}^{V}\frac{1}{\omega \left(v\right)}=1,\{\omega \left(v\right){\}}_{v=1}^{V}⪰0. \end{array} \end{array}$$ (10)

To capture the complex similarity relationships among cells, we introduce second-order information. For the *v*-th omic, let ${𝒩}_{i}^{\alpha }(v)$ denote the α-nearest neighbors of cell *i*, and $𝐍\left(v\right)\in \{0,1{\}}^{n\times n}$ represent the second-order co-occurrence matrix where elements are determined by:


$$\begin{array}{c} \begin{array}{c} n_{ij}\left(v\right)=\left\{ \begin{array}{cc} 1, & \text{If }j\in {𝒩}_{i}^{\alpha }(v)\text{ and }|{𝒩}_{i}^{\alpha }(v)\cap {𝒩}_{j}^{\alpha }(v)|\geq \beta ,\\ 0, & \text{else.} \end{array}\right. \end{array} \end{array}$$ (11)

**Fig 19:**
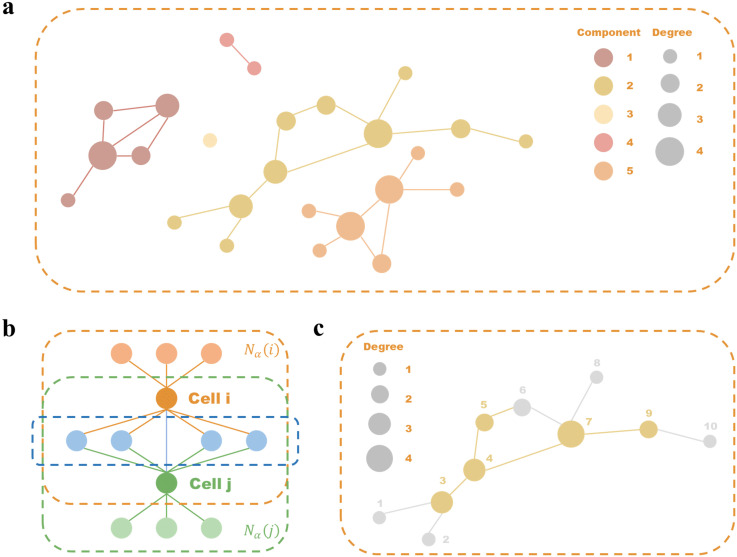
Construction of supercells. **(a)** Example graph constructed from adjacency matrix **M**. **(b)** Illustration of second-order co-occurrence neighbors based on shared α-nearest neighbors exceeding threshold β. **(c)** Example of probabilistic pruning within a connected component.

An example of second-order co-occurrence neighbors is visualized in Fig 19b.

The cross-omics consistency matrix $𝐌=(m_{ij}{)}_{n\times n}\in \{0,1{\}}^{n\times n}$ identifies persistent neighborhood relationships across modalities:


$$\begin{array}{c} \begin{array}{c} m_{ij}=\left\{ \begin{array}{cc} 1, & \text{If }\sum_{v=1}^{V}n_{ij}\left(v\right)>\left\lfloor \frac{V}{2}\right\rfloor ,\\ 0, & \text{else}. \end{array}\right. \end{array} \end{array}$$ (12)

Using the consistency matrix **M** as an adjacency matrix, we construct the multi-omics similarity graph ${𝒢}_{M}$ in Fig 19a. Let $\left\{{𝒢}_{M}(1),{𝒢}_{M}(2),...,{𝒢}_{M}(L)\right\}$ denote the *L* connected components of ${𝒢}_{M}$. While the connectedness principle suggests clustering co-component cells together, boundary cells with weak connectedness to other intra-component cells require special treatment. Therefore, we define cell’s higher‑order neighborhood as the full set of cells that belong to the same connected components. We compute each cell’s degree centrality and then probabilistically discard cells whose profiles deviate from the group consensus.

For each component ${𝒢}_{M}(l)$ (l∈[1,L]), compute degree centrality of cell *i*:


$$\begin{array}{c} \begin{array}{c} {DC}_{i}\left(l\right)=\frac{\deg(i)}{|{𝒢}_{M}(l)|-1},i\in {𝒢}_{M}(l) \end{array} \end{array}$$ (13)

where $\left|{𝒢}_{M}(l)\right|$ denotes component cardinality.

The high-order similarity metric of cell *i* in the *l*-th connected component is then:


$$\begin{array}{c} \begin{array}{c} {HS}_{i}\left(l\right)=\left\{ \begin{array}{cc} \frac{{DC}_{i}(l)-\min_{j}{DC}_{j}(l)}{\max_{j}{DC}_{j}(l)-\min_{j}{DC}_{j}(l)}, & \text{If }i\in {𝒢}_{M}(l)\text{ and }|{𝒢}_{M}(l)|\geq 3,\\ 1, & \text{else.} \end{array}\right. \end{array} \end{array}$$ (14)

The cell elimination probability *P*_*i*_(*l*) follows:


$$\begin{array}{c} \begin{array}{c} P_{i}\left(l\right)=1-{HS}_{i}\left(l\right). \end{array} \end{array}$$ (15)

An iterative pruning process is implemented where cell *i* is removed if $U_{i}\leq P_{i}\left(l\right)$ for $U_{i}\sim \text{Uniform}(0,1)$. Fig 19c demonstrates this workflow.

Each pruned component and eliminated cell is regarded as a “supercell,” which is a element in the set $\{SC(s)\overset{S}{\underset{s=1}{\}}}$.

Then each cluster ${𝒞}_{p}$ becomes ${⋃}_{s\in {ℐ}_{p}}SC(s)$ where ${ℐ}_{p}\subseteq \{1,...,S\}$. The similarity between ${𝒞}_{p}$ and $\bar{{𝒞}_{p}}$ in the *v*-th omic is reformulated as:


$$\begin{array}{c} \begin{array}{c} \begin{array}{cc} sim({𝒞}_{p},\bar{{𝒞}_{p}}) & =\sum_{i\in {𝒞}_{p}}\sum_{j\in \bar{{𝒞}_{p}}}(a_{ij}(v)+a_{ji}(v))\\ & =\sum_{y\in I_{p}}\sum_{z\in \bar{I_{p}}}\sum_{i\in SC(y)}\sum_{j\in SC(z)}(a_{ij}(v)+a_{ji}(v))\\ & =\sum_{y\in I_{p}}\sum_{z\in \bar{I_{p}}}as_{yz}(v), \end{array} \end{array} \end{array}$$ (16)

where the similarity between supercells is:


$$\begin{array}{c} \begin{array}{c} as_{yz}(v)=\sum_{i\in SC(y)}\sum_{j\in SC(z)}(a_{ij}(v)+a_{ji}(v)). \end{array} \end{array}$$ (17)

Then, the optimization framework transitions to:


$$\begin{array}{c} \begin{array}{c} \min_{\{I_{p}\overset{C}{\underset{p=1}{\}}},\{\omega \left(v\right)\overset{V}{\underset{v=1}{\}}}}\sum_{v=1}^{V}\omega (v)\sum_{p=1}^{C}\sum_{y\in I_{p}}\sum_{z\in \bar{I_{p}}}as_{yz}(v),\;s.t.{𝒞}_{p}={⋃}_{s\in I_{p}}SC(s),\overset{C}{\underset{p=1}{⋃}}{𝒞}_{p}=𝒞,\;\forall p\neq q\in {\mathbb{Z}}_{\left[1,C\right]},I_{p}⋂I_{q}=\emptyset ,\;\forall v\in {\mathbb{Z}}_{\left[1,V\right]},\sum_{v=1}^{V}\frac{1}{\omega \left(v\right)}=1,\{\omega \left(v\right)\overset{V}{\underset{v=1}{\}}}⪰0. \end{array} \end{array}$$ (18)

Further, the matrix formulation using cluster indicator matrix $𝐄\in \{0,1{\}}^{S\times C}$ is:


$$\begin{array}{c} \begin{array}{c} \min_{{𝐄}_{S\times C},\{\omega \left(v\right)\overset{V}{\underset{v=1}{\}}}}\sum_{v=1}^{V}\omega \left(v\right)\sum_{p=1}^{C}\|𝐋\left(v\right){𝐞}_{p}{\|}_{1}\;s.t.\sum_{v=1}^{V}\frac{1}{\omega \left(v\right)}=1,\{\omega \left(v\right)\overset{V}{\underset{v=1}{\}}}⪰0,\;𝐄\in \{0,1{\}}_{S\times C},E1=1, \end{array} \end{array}$$ (19)

where ${𝐄}_{S\times C}=(e_{1},e_{2},...,e_{C})$ represents the clustering result of supercells, 𝐋(v) is the Laplacian matrix of $AS\left(v\right)=(as_{yz}\left(v\right))$ defined in Eq. (17).

We implement a block coordinate descent (BCD) optimization framework with alternating updates between cluster assignments ${𝐄}_{S\times C}$ and modality weights $\{\omega \left(v\right)\overset{V}{\underset{v=1}{\}}}$. The iterative scheme proceeds as follows:

Firstly, given current cluster indicators **E**^(*t*)^, the analytical solution of $\{\omega^{\left(t+1\right)}\left(v\right)\overset{V}{\underset{v=1}{\}}}$ can be obtained as Eq. (20).


$$\begin{array}{c} \begin{array}{c} \omega^{\left(t+1\right)}\left(v\right)=\frac{\sum_{v^{\prime }=1}^{V}\sqrt{\epsilon^{\left(t+1\right)}\left(v^{\prime }\right)}}{\sqrt{\epsilon^{\left(t+1\right)}\left(v\right)}},\forall v\;\epsilon^{\left(t+1\right)}\left(v\right)=\sum_{p=1}^{C}\|𝐋\left(v\right){𝐞}_{p}^{\left(t\right)}{\|}_{1}. \end{array} \end{array}$$ (20)

Secondly, fix $\{\omega \left(v\right)\overset{V}{\underset{v=1}{\}}}$ and update ${𝐄}_{S\times C}$. We define the matrix $\hat{L}$ as Eq. (21).


$$\begin{array}{c} \begin{array}{c} \hat{L}=\sum_{v=1}^{V}\omega \left(v\right)(𝐋\left(v\right)+(𝐋\left(v\right){)}^{T}). \end{array} \end{array}$$ (21)

Meanwhile, define ${𝐞}_{1\times C}^{\left[i\right]}$ as the row vector where the *i*-th element is 1 and the remaining elements are 0, ${𝐞}_{1\times C}^{\left[0\right]}$ as a zero vector, and $E^{\left[i\right]}$ as a matrix with ${𝐞}^{\left[i\right]}$ as the *d*-th row and the remaining rows being the same as **E**.

Next, we traverse the number of rows *d* and update the *d*-th row. Our objective function is:


$$\begin{array}{c} \begin{array}{c} y=\underset{i\in {\mathbb{Z}}_{\left[1,C\right]}}{**argmin}\sum_{p=1}^{C}({𝐞}_{p}^{\left[i\right]}{)}^{T}\hat{𝐋}{𝐞}_{p}^{\left[i\right]}. \end{array} \end{array}$$ (22)

If we define:


$$\begin{array}{c} \begin{array}{c} {ℒ}_{i,p}=({𝐞}_{p}^{\left[i\right]}{)}^{T}\hat{𝐋}{𝐞}_{p}^{\left[i\right]},i\in {\mathbb{Z}}_{\left[0,C\right]},p\in {\mathbb{Z}}_{\left[1,C\right]}, \end{array} \end{array}$$ (23)

then the objective function is transformed into:


$$\begin{array}{c} \begin{array}{c} y=\underset{i\in {\mathbb{Z}}_{\left[1,C\right]}}{**argmin}{ℒ}_{i,i}-{ℒ}_{0,i}. \end{array} \end{array}$$ (24)

Find the index in the *d*-th row vector **e**^*d*^ where the element is 1 and denote it as *j*. Then, calculate ${ℒ}_{i,i}-{ℒ}_{0,i}$ based on Eq. (25).


$$\begin{array}{c} \begin{array}{c} {ℒ}_{i,i}-{ℒ}_{0,i}=\left\{ \begin{array}{cc} 2{𝐞}_{i}^{T}{\hat{𝐥}}_{d}-{\hat{l}}_{d,d}, & \text{If }i=j,\\ 2{𝐞}_{i}^{T}{\hat{𝐥}}_{d}+{\hat{l}}_{d,d}, & \text{If }i\neq j. \end{array}\right. \end{array} \end{array}$$ (25)

When traversing all ${ℒ}_{i,i}-{ℒ}_{0,i}$ corresponding to *i*, the optimal solution *y* can be found, that is, the optimal solution in row *d*:


$$\begin{array}{c} \begin{array}{c} {\bar{𝐞}}^{d}=\delta (y,C), \end{array} \end{array}$$ (26)

where δ(y,C) is a row vector where the *y*-th element is 1 and the remaining elements are 0.

Alternate update ${𝐄}_{S\times C}$ and $\{\omega \left(v\right)\overset{V}{\underset{v=1}{\}}}$ until convergence. The terminal cluster assignments are obtained by mapping **E** to supercell $\{SC(s)\overset{S}{\underset{s=1}{\}}}$.

In the experiments, the cluster indicator matrix **E** is initialized using the FINCH algorithm [47]. The optimization terminates when the relative change of the objective value between two successive iterations falls below 10^−20^ after the first 10 iterations, or when the maximum number of iterations (50) is reached.

**Algorithm 1 The Algorithm of scHG**

**Require:** $\{𝐗(v)\overset{V}{\underset{v=1}{\}}}$

**Ensure:** $\{\omega (v)\overset{V}{\underset{v=1}{\}}},𝐄$

1: Calculate $\{𝐀(v)\overset{V}{\underset{v=1}{\}}}$ via (6).

2: Calculate $\{SC(s)\overset{S}{\underset{s=1}{\}}}$ via (11) to (15).

3: Calculate $\{AS(v)\in {\mathbb{R}}^{S\times S}\overset{V}{\underset{v=1}{\}}}$ via (17).

4: Initialize **E**.

5: **while** not converge **do**

6:   update $\{\omega (v)\overset{V}{\underset{v=1}{\}}}$ via (20).

7:   Calculate $\hat{L}$ via (21).

8:   **while** not converge and d∈[1,S] **do**

9:     Find the index of 1 in ed and save as j.

10:     Calculate $y=\underset{i\in {\mathbb{Z}}_{\left[1,C\right]}}{**argmin}{ℒ}_{i,i}-{ℒ}_{0,i}$ via (25) and get ${\bar{𝐞}}^{d}=\delta (y,C)$.

11:     d⇐d+1

12:   **end while**

13: **end while**

### 5.3 Estimation of the cluster numbers

When the true cluster number is unknown, we propose a consensus estimation framework for multi-omics data $\{𝐗\left(v\right)\overset{V}{\underset{v=1}{\}}}$. Each omic dataset is independently subjected to *v*-means clustering with elbow method optimization. For dataset **X**(*v*), the within-cluster sum of squared errors (SSE) for *C*(*v*) clusters is defined as:


$$\begin{array}{c} \begin{array}{c} SSE_{C(v)}=\sum_{p=1}^{C(v)}\sum_{i\in {𝒞}_{p}}\|{𝐱}^{i}\left(v\right)-\mu_{p}(v){\|}^{2},\;\mu_{p}(v)=\frac{1}{\left|{𝒞}_{p}\right|}\sum_{i\in {𝒞}_{p}}{𝐱}^{i}\left(v\right), \end{array} \end{array}$$ (27)

where ${𝒞}_{p}$ denotes the *p*-th cluster cell set.

The optimal cluster number *C*(*v*) per omic is determined by maximizing the elbow criterion:


$$\begin{array}{c} \begin{array}{c} C(v)=\underset{C(v)}{\mathrm{\backslash arg}\max}\{\frac{SSE_{C(v)}-SSE_{C-1(v)}}{SSE_{C+1(v)}-SSE_{C(v)}}\}+1. \end{array} \end{array}$$ (28)

When omic-specific estimates $\{C\left(v\right)\overset{V}{\underset{v=1}{\}}}$ identical, the global cluster number *C* adopts their consensus value. For discrepancy cases, we compute the fused high-order multi-view similarity matrix $\tilde{A}$ and its optimal $C(\tilde{A})$ using analogous criteria (Eq. (27)- (28)), then determine:


$$\begin{array}{c} \begin{array}{c} C=\text{round}(\alpha_{1}C(1)+\ldots +\alpha_{V}C(V)+\alpha_{\tilde{A}}C(\tilde{A})), \end{array} \end{array}$$ (29)

where $\alpha_{v}$ and $\alpha_{\tilde{A}}$ are weight coefficients.

### 5.4 Time complexity analysis

Supercell constructing includes Eq. (11) to Eq. (15) executing inO(n+|ℰ|) time, where *n* and |ℰ| denote vertex and edge counts respectively. Supercell clustering optimization requires *O*(*S*^2*VT*^) operations, with *S* supercells, *V* omics, and *T* iterations. So the total complexity $O(n+|ℰ|+S^{2}VT)$ simplifies to $O(n+|ℰ|+S^{2})$ given V≪S. Since $|ℰ|\ll n^{2}$ and S≪n empirically, the effective complexity stays well below the quadratic upper bound *O*(*n*^2^).

### 5.5 Datasets and preprocessing

We utilized five real-world datasets and one simulated dataset as shown in Table 11, which are elaborated as follows:

(1) PBMC10× dataset: The 10× dataset contains 6661 cells with 7 cell types. The data were extracted from [48].
(2) PBMC_Inhouse dataset: The PBMC_Inhouse dataset contains 1182 cells with 6 cell types. The data were extracted from [12].
(3) Bmcite dataset: The Bmcite dataset contains 30672 cells with 27 cell types. The data were extracted from [49].
(4) mESC dataset: The mESC dataset contains 77 cells with 2 cell types. The data were extracted from [50]. For each omics, we select the 125 columns of features with the largest variance.
(5) PBMC_Cao dataset: The PBMC_Cao dataset contains 8185 cells with 9 cell types. The data were extracted from [51].
(6) Sim dataset: The Sim dataset contains 500 cells with 4 cell types. The data were extracted from [10].

**Table 11:** Detailed information of single-cell multi-omics datasets.

Datasets	Cell	RNA	ADT	ATAC	Type	Refs
PBMC10×	6661	500	14		7	ref [48]
PBMC_Inhouse	1182	33538	10		6	ref [12]
Bmcite	30672	17009	25		27	ref [49]
mESC	77	15000		5000	2	ref [50]
PBMC_Cao	8185	500		500	9	ref [51]
Sim	500	5000		2000	4	ref [10]

For datasets where any omics modality contains ≥5000 features, we perform variance-based feature selection to retain the top 125 highest-variance features. This preprocessing ensures computational efficiency while preserving biologically informative signals.

### 5.6 Compared methods

**GBS [15]** GBS is a graph-based approach for Multi-View Clustering by proposing a unified framework studying generalization and graph metric impact. Its novel method effectively constructs adaptive graph matrices, automatically weights them, and directly produces final clusters. The code and datasets are released at: https://github.com/cswanghao/gbs.

**scAI [10]** scAI is a single-cell aggregation and integration method designed to deconvolute cellular heterogeneity from parallel transcriptomic and epigenomic profiles by iteratively learning and aggregating sparse epigenomic signals across similar cells in an unsupervised manner, enabling coherent fusion with transcriptomics to dissect multi-omic heterogeneity and uncover regulatory mechanisms. The code and datasets are released at: https://github.com/amsszlh/scAI.

**SMSC [7]** SMSC unifies joint nonnegative-spectral embedding with two distinctive features: (1) nonnegative embeddings directly yield cluster assignments, eliminating post-processing steps; (2) automatic parameter learning removes manual tuning requirements. The code and datasets are released at: https://github.com/sudalvxin/SMSC.

**CGD [16]** CGD proposes a parameter-free, convergence-guaranteed approach for multi-view clustering by cross-view graph diffusion. It addresses key limitations of existing methods—model dependency, high computational complexity, and hyperparameter sensitivity—through an iterative diffusion process that 1) refines single-view graphs by preserving manifold structures and 2) leverages complementary information across views, yielding a unified graph that significantly outperforms benchmarks on seven evaluation metrics. The code and datasets are released at: https://github.com/ChangTang/CGD.

**scMNMF [13]** scMNMF is an algorithm that jointly performs dimensionality reduction and cell clustering through non-negative matrix factorization (NMF) on single-cell multi-omics data. The code and datasets are released at: https://github.com/yushanqiu/scMNMF.

**GSTRPCA [52]** GSTRPCA is an adaptive tensor decomposition framework extending Tensor Robust Principal Component Analysis (TRPCA). It incorporates low-rank and sparse constraints via a weighted thresholding scheme, preserving data structure integrity while extracting latent cross-omic features. The code and datasets are released at: https://github.com/GGL-B/GSTRPCA.

For all baseline methods, we used the official implementations released by the original authors and followed the recommended settings in their respective papers. Unless otherwise specified, all hyperparameters were kept at their default values to ensure fair comparisons and reproducibility.

### 5.7 Hyperparameter configuration

The similarity matrix **A**(*v*) (Eq. (6)) employs adaptive regularization:


$$\begin{array}{c} \begin{array}{c} \gamma =\left\{ \begin{array}{cc} 10, & \text{if }\max_{v}\{m_{v}\}<5000,\\ 20, & \text{otherwise}. \end{array}\right. \end{array} \end{array}$$ (30)

For second-order co-occurrence matrix **N**(*v*) (Eq. (11)), we fix α=5 and β=2. Cluster number estimation (Eq. (29)) uses modality-weighted fusion:


$$\begin{array}{c} \begin{array}{c} C=\text{round}\left(\frac{2}{9}C\left(1\right)+\frac{3}{9}C\left(2\right)+\frac{4}{9}C\left(\tilde{A}\right)\right) \end{array} \end{array}$$ (31)

where weights $\left(\frac{2}{9},\frac{3}{9},\frac{4}{9}\right)$ reflect the relative importance of RNA, ADT(or ATAC), and fused similarity features respectively.

All experiments were executed on a Lenovo Legion R7000 2020 laptop equipped with an AMD Ryzen 7 4800H processor and 16 GB 3200 MT/s RAM, using MATLAB R2022a.

### 5.8 Performance metrics

Two established metrics are employed to assess clustering quality: Adjusted Rand Index (**ARI**) and Normalized Mutual Information (**NMI**). Consider two partitions: the ground truth partition $𝒯=\{{𝒯}_{1},{𝒯}_{2},...,{𝒯}_{t}\}$ and the clustered partition $𝒞=\{{𝒞}_{1},{𝒞}_{2},...,{𝒞}_{p}\}$, where *t* and *p* denote the cardinality of true and clustered categories, respectively.

The **ARI** is formulated as:


ARI(𝒞,𝒯)=∑ij(nij2)−[∑i(ai2)∑j(bj2)]/(n2)12[∑i(ai2)+∑j(bj2)]−[∑i(ai2)∑j(bj2)]/(n2), (32)

where *n*_*ij*_ denotes the overlap between ${𝒯}_{i}$ and ${𝒞}_{j}$, $a_{i}=|{𝒯}_{i}|$, $b_{j}=|{𝒞}_{j}|$, and *n* is the total sample size. **ARI** ranges [-1, 1], where 1 indicates a perfect match for the reference label.

The **NMI** is defined as:


$$\begin{array}{c} \begin{array}{c} \text{NMI}(𝒞,𝒯)=\frac{2\sum_{ij}n_{ij}\log\frac{nn_{ij}}{a_{i}b_{j}}}{-\sum_{i}a_{i}\log\frac{a_{i}}{n}+\sum_{j}b_{j}\log\frac{b_{j}}{n}}. \end{array} \end{array}$$ (33)

**NMI** ranges [0,1], where 1 indicates full information sharing.

Note that our ablation analysis further incorporated six validation metrics: Accuracy (**ACC**; [40]), **Purity** ([43]), **F1-score** ([44]), **Precision** ([44]), **Recall** ([44]), and Rand Index (**RI**; [45]).

## Supporting information

S1 TableCluster-specific marker lists (genes and proteins) for the PBMC10× dataset.For each predicted cluster, we report features identified by one-sided Welch’s *t*-tests (cluster vs. rest), including the nominal one-sided *p*-value, $\log_{2}FC$, and the corresponding cluster index.(XLSX)

S2 Table Gene Ontology (GO) enrichment results for selected marker sets. For each enriched term, we report the ontology category (BP/CC/MF), GO identifier and description, enrichment ratios, nominal and adjusted significance values (e.g., *p*-value, *p*-adjusted, and *q*-value), the contributing gene list, and related summary statistics.
(XLSX)

S3 Table Ablation and enrichment analyses for Cluster 6 and the associated representative supercell (Supercell 62). This file includes differential marker genes/proteins for Cluster 6, Supercell 62, and the corresponding “Cluster 6 minus Supercell 62” setting, together with pathway enrichment outputs (GSEA) across GO, KEGG, MSigDB, and Reactome.
(XLSX)

S4 Table Ablation analysis for Cluster 5 and the associated representative supercell (Supercell 363). This file reports differential marker genes/proteins for Cluster 5, Supercell 363, and the corresponding “Cluster 5 minus Supercell 363” setting.
(XLSX)

S5 Table Detailed prediction and supercell-graph outputs used in downstream evaluations. This file provides the confusion matrix, per-cell ground-truth and predicted labels (including pruned variants), cell-to-supercell assignments and supercell counts, and graph statistics for Supercell 62 (adjacency matrix and degree centrality).
(XLSX)

S6 Table It summarizes the four largest supercells identified in the *Pbmc_Inhouse* dataset. For each supercell, marker proteins and marker genes were identified through hypothesis testing.
(XLSX)

S1 AppendixSupplementary materials associated with this study.Fig A shows the comparative t-SNE visualization of latent representations on the mESC dataset. Fig B shows the comparative t-SNE visualization of latent representations on the PBMC_Inhouse dataset. Fig C shows the comparative t-SNE visualization of latent representations on the Sim dataset. Fig D shows the comparative t-SNE visualization of latent representations on the PBMC_Cao dataset. Algorithm section provides the detailed pseudo-code of the scHG framework, including the full optimization pipeline. In addition, this appendix includes sensitivity analyses for key components, including cluster number selection, hyperparameters (α, β), similarity threshold (γ), and supercell construction strategy, as well as a summary table of all hyperparameters and their default values.(PDF)
