# Supplementary material for: scHG: A supercell framework with high-order graph learning enables scalable multi-omics analysis
Source: PLoS Comput Biol. 2026 May 6;22(5):e1013851. doi: 10.1371/journal.pcbi.1013851 (PMC13167035; doi:10.1371/journal.pcbi.1013851)
Supplement: S1 Appendix — Fig A shows the comparative t-SNE visualization of latent representations on the mESC dataset. Fig B shows the comparative t-SNE visualization of latent representations on the PBMC_Inhouse dataset. Fig C shows the comparative t-SNE visualization of latent representations on the Sim dataset. Fig D shows the comparative t-SNE visualization of latent representations on the PBMC_Cao dataset. Algorithm section provides the detailed pseudo-code of the scHG framework, including the full optimization pipeline. In addition, this appendix includes sensitivity analyses for key components, including cluster number selection, hyperparameters (α, β), similarity threshold (γ), and supercell construction strategy, as well as a summary table of all hyperparameters and their default values. (PDF) [file pcbi.1013851.s007.pdf]

Supplementary Information

1 Figures

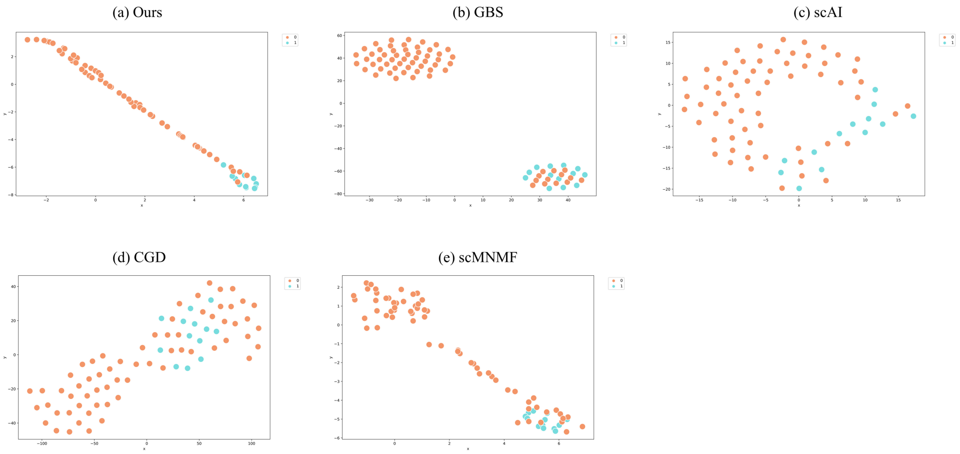

Fig A. Comparative t-SNE Visualization of Latent Representations on mESC Dataset: (a) scHG, (b) GBS, (c) scAI, (d) CGD, (e) scMNMF.

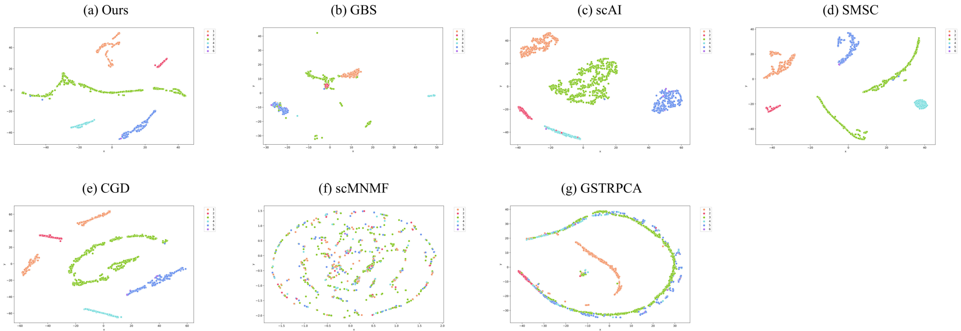

Fig B. Comparative t-SNE Visualization of Latent Representations on PBMC_Inhouse Dataset: (a) scHG, (b) GBS, (c) scAI, (d) CGD, (e) scMNMF.

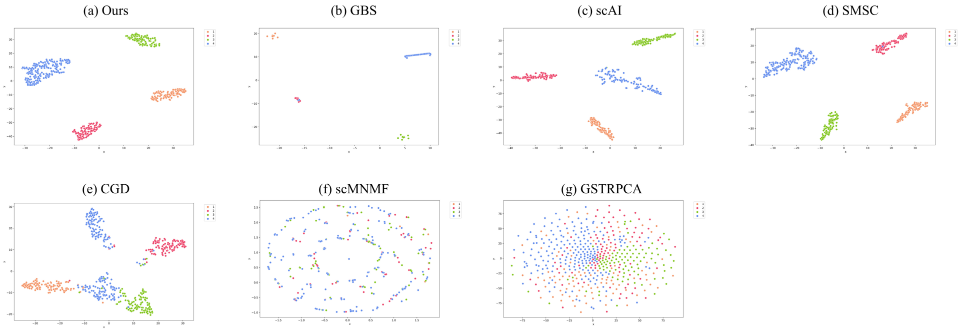

Fig C. Comparative t-SNE Visualization of Latent Representations on Sim Dataset: (a) scHG, (b) GBS, (c) scAI, (d) CGD, (e) scMNMF.

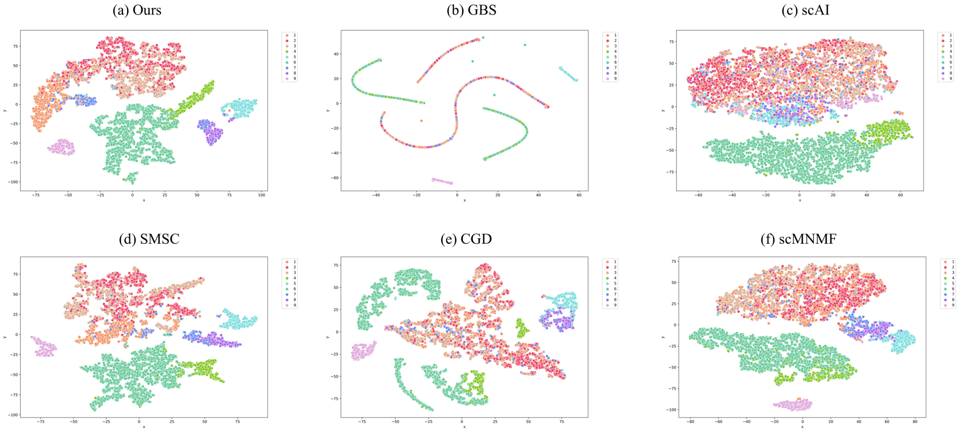

Fig D. Comparative t-SNE Visualization of Latent Representations on PBMC_Cao Dataset: (a) scHG, (b) GBS, (c) scAI, (d) CGD, (e) scMNMF.

2 Algorithm

2.1 Pseudo-code for scHG

Consider a collection of V multi-omics datasets $\{\mathbf{X}(v)\}_{v=1}^{V}$, where each matrix $\mathbf{X}(v) \in \mathbb{R}^{n \times m_v}$ represents a distinct omics data. Here n denotes the number of cells, and m_v the number of features for the v-th omics data $\mathbf{X}(v)$. Let $\mathbf{x}^i(v)$ denote the i-th row vector (i-th cell) and $\mathbf{x}_j(v)$ the j-th column vector (j-th feature) of $\mathbf{X}(v)$, corresponding to biological features such as gene expression (RNA-seq), surface protein abundance (antibody-derived tags (ADT)), or chromatin accessibility (ATAC-seq).

In traditional omics clustering analyses, similarity between cells is frequently quantified using Euclidean distance, whereby smaller distances indicate greater similarity. In contrast, Eq. (1) defines an alternative angle-aware metric: the Pearson correlation coefficient (PCC) between cells.

$$\mathbf{PCC}\left(\mathbf{x}^i(v), \mathbf{x}^j(v)\right) = \frac{\left(\mathbf{x}^i(v) - \bar{\mathbf{x}}^i(v)\right)^T \left(\mathbf{x}^j(v) - \bar{\mathbf{x}}^j(v)\right)}{\|\mathbf{x}^i(v) - \bar{\mathbf{x}}^i(v)\|_2 \|\mathbf{x}^j(v) - \bar{\mathbf{x}}^j(v)\|_2}, \tag{1}$$

Where $\mathbf{x}^i(v)$ and $\mathbf{x}^j(v)$ are any two cells in the v-th omics, respectively. Notably, the Pearson correlation coefficient can be interpreted as the cosine of the angle between mean-centered feature vectors, which makes the similarity invariant to scale differences across cells.

Given these comparative advantages of the angle-aware metric over Euclidean distance, our algorithm employs angle-aware metric as the primary similarity measure. The effectiveness of this design choice is further validated through ablation experiments, where replacing the angle-aware metric with Euclidean distance leads to consistent performance degradation across several datasets.

The cell similarity matrix $\mathbf{A}(v) = (a_{ij})_{n \times n}$ for the v-th omic is constructed using the γ-proximity linear similarity metric. Specifically, we first compute the Pearson correlation matrix $\mathbf{PCC}(v)$ of matrix $\mathbf{X}(v)$ by:

$$\mathbf{PCC}(v)_{ij} = \mathbf{PCC}\left(\mathbf{x}^i(v), \mathbf{x}^j(v)\right) \tag{2}$$

Then for each cell i, we extract its γ+1 nearest neighbors (excluding self) from $\mathbf{PCC}(v)$ to form the truncated ordered distance matrix $\mathbf{P\tilde{C}C}(v)$, yielding the similarity coefficients:

$$a_{ij}(v) = \begin{cases} \frac{\mathbf{P\tilde{C}C}(v)_{i(\gamma+1)} - \mathbf{P\tilde{C}C}(v)_{ij}}{\gamma \cdot \mathbf{P\tilde{C}C}(v)_{i(\gamma+1)} - \sum_{j=1}^{\gamma} \mathbf{P\tilde{C}C}'(v)_{ij}}, & \text{If } 1 \leq j \leq \gamma, \\ 0, & \text{else.} \end{cases} \tag{3}$$

Given C cell clusters, let $\mathcal{C}_p$ denote the p-th cluster. The inter-cluster similarity between $\mathcal{C}_p$ and its complement $\overline{\mathcal{C}_p}$ is defined as:

$$sim(\mathcal{C}_p, \bar{\mathcal{C}_p}) = \sum_{i \in \mathcal{C}_p} \sum_{j \in \bar{\mathcal{C}_p}} (a_{ij}(v) + a_{ji}(v)). \tag{4}$$

The total inter-cluster similarities across all clusters and their complements is quantified as:

$$\sum_{p=1}^{C} sim(\mathcal{C}_p, \bar{\mathcal{C}_p}) = \sum_{p=1}^{C} \sum_{i \in \mathcal{C}_p} \sum_{j \in \bar{\mathcal{C}_p}} (a_{ij}(v) + a_{ji}(v)). \tag{5}$$

To account for omics-specific contributions, we introduce modality weights $\{\omega(v)\}_{v=1}^{V}$, yielding the weighted multi-omics similarity:

$$\sum_{v=1}^{V} \omega(v) \sum_{p=1}^{C} \sum_{i \in \mathcal{C}_p} \sum_{j \in \bar{\mathcal{C}_p}} (a_{ij}(v) + a_{ji}(v)). \tag{6}$$

To seek maximally separable partitions, the total multi-omics similarity should be minimized through the following constrained optimization:

$$
\begin{aligned}
&\min_{\{\mathcal{C}_p\}_{p=1}^{C},\{\omega(v)\}_{v=1}^{V}} \sum_{v=1}^{V} \omega(v) \sum_{p=1}^{C} \sum_{i\in\mathcal{C}_p} \sum_{j\in\bar{\mathcal{C}}_p} (a_{ij}(v)+a_{ji}(v)), \\
&\text{s.t.} \bigcup_{p=1}^{C} \mathcal{C}_p = \mathcal{C}, \forall p \neq q \in \mathbb{Z}_{[1,C]}, \mathcal{C}_p \bigcap \mathcal{C}_q = \varnothing, \\
&\forall v \in \mathbb{Z}_{[1,V]}, \sum_{v=1}^{V} \frac{1}{\omega(v)} = 1, \{\omega(v)\}_{v=1}^{V} \succeq 0.
\end{aligned}
\tag{7}
$$

To capture the complex similarity relationships among cells, we introduce second-order information. For the v-th omic, let $\mathcal{N}_i^{\alpha}(v)$ denote the α-nearest neighbors of cell i, and $\mathbf{N}(v) \in \{0,1\}^{n\times n}$ represent the second-order co-occurrence matrix where elements are determined by:

$$
n_{ij}(v) = \begin{cases} 1, & \text{If } j \in \mathcal{N}_i^{\alpha}(v) \text{ and } |\mathcal{N}_i^{\alpha}(v) \cap \mathcal{N}_j^{\alpha}(v)| \geq \beta, \\ 0, & \text{else.} \end{cases} \tag{8}
$$

The cross-omics consistency matrix $\mathbf{M} = (m_{ij})_{n\times n} \in \{0,1\}^{n\times n}$ identifies persistent neighborhood relationships across modalities:

$$
m_{ij} = \begin{cases} 1, & \text{If } \sum_{v=1}^{V} n_{ij}(v) > \left\lfloor \frac{V}{2} \right\rfloor, \\ 0, & \text{else.} \end{cases} \tag{9}
$$

Using the consistency matrix $\mathbf{M}$ as an adjacency matrix, we construct the multi-omics similarity graph $\mathcal{G}_M$. Let $\{\mathcal{G}_M(1), \mathcal{G}_M(2), ..., \mathcal{G}_M(L)\}$ denote the L connected components of $\mathcal{G}_M$. While the connectedness principle suggests clustering co-component cells together, boundary cells with weak connectedness to other intra-component cells require special treatment. Therefore, we define cell's higher-order neighborhood as the full set of cells that belong to the same connected components. We compute each cell's degree centrality and then probabilistically discard cells whose profiles deviate from the group consensus.

For each component $\mathcal{G}_M(l)$ ($l \in [1, L]$), compute degree centrality of cell i:

$$
\mathbf{DC}_i(l) = \frac{\deg(i)}{|\mathcal{G}_M(l)| - 1}, i \in \mathcal{G}_M(l) \tag{10}
$$

where $|\mathcal{G}_M(l)|$ denotes component cardinality.

The high-order similarity metric of cell i in the l-th connected component is then:

$$
\mathbf{HS}_i(l) = \begin{cases} \frac{\mathbf{DC}_i(l) - \min\limits_j \mathbf{DC}_j(l)}{\max\limits_j \mathbf{DC}_j(l) - \min\limits_j \mathbf{DC}_j(l)}, & \text{If } i \in \mathcal{G}_M(l) \text{ and } |\mathcal{G}_M(l)| \geq 3, \\ 1, & \text{else.} \end{cases} \tag{11}
$$

The cell elimination probability $P_i(l)$ follows:

$$
P_i(l) = 1 - \mathbf{HS}_i(l). \tag{12}
$$

An iterative pruning process is implemented where cell i is removed if $U_i \leq P_i(l)$ for $U_i \sim \text{Uniform}(0,1)$.

Each pruned component and eliminated cell is regarded as a "supercell", which is a element in the set $\{SC(s)\}_{s=1}^{S}$.

Then each cluster $\mathcal{C}_p$ becomes $\bigcup_{s\in\mathcal{I}_p} SC(s)$ where $\mathcal{I}_p \subseteq \{1, ..., S\}$. The similarity between $\mathcal{C}_p$ and $\bar{\mathcal{C}_p}$ in the v-th omic is reformulated as:

$$\begin{aligned} sim(\mathcal{C}_p, \bar{\mathcal{C}_p}) &= \sum_{i\in\mathcal{C}_p} \sum_{j\in\bar{\mathcal{C}_p}} (a_{ij}(v) + a_{ji}(v)) \\ &= \sum_{y\in I_p} \sum_{z\in\bar{I_p}} \sum_{i\in SC(y)} \sum_{j\in SC(z)} (a_{ij}(v) + a_{ji}(v)) \\ &= \sum_{y\in I_p} \sum_{z\in\bar{I_p}} as_{yz}(v), \end{aligned} \tag{13}$$

where the similarity between supercells is:

$$as_{yz}(v) = \sum_{i\in SC(y)} \sum_{j\in SC(z)} (a_{ij}(v) + a_{ji}(v)). \tag{14}$$

Then, the optimization framework transitions to:

$$\begin{gathered} \min_{\{I_p\}_{p=1}^C, \{\omega(v)\}_{v=1}^V} \sum_{v=1}^V \omega(v) \sum_{p=1}^C \sum_{y\in I_p} \sum_{z\in\bar{I_p}} as_{yz}(v), \\ \text{s.t.} \mathcal{C}_p = \bigcup_{s\in I_p} SC(s), \bigcup_{p=1}^C \mathcal{C}_p = \mathcal{C}, \\ \forall p \neq q \in \mathbb{Z}_{[1,C]}, I_p \bigcap I_q = \varnothing, \\ \forall v \in \mathbb{Z}_{[1,V]}, \sum_{v=1}^V \frac{1}{\omega(v)} = 1, \{\omega(v)\}_{v=1}^V \succeq 0. \end{gathered} \tag{15}$$

Further, the matrix formulation using cluster indicator matrix $\mathbf{E} \in \{0,1\}^{S\times C}$ is:

$$\begin{gathered} \min_{\mathbf{E}_{S\times C}, \{\omega(v)\}_{v=1}^V} \sum_{v=1}^V \omega(v) \sum_{p=1}^C \|\mathbf{L}(v)\mathbf{e}_p\|_1 \\ \text{s.t.} \sum_{v=1}^V \frac{1}{\omega(v)} = 1, \{\omega(v)\}_{v=1}^V \succeq 0, \\ \mathbf{E} \in \{0,1\}_{S\times C}, \mathbf{E}\mathbf{1} = \mathbf{1}, \end{gathered} \tag{16}$$

where $\mathbf{E}_{S\times C} = (e_1, e_2, ..., e_C)$ represents the clustering result of supercells, $\mathbf{L}(v)$ is the Laplacian matrix of $\mathbf{AS}(v) = (as_{yz}(v))$ defined in Eq. (14).

We implement a block coordinate descent (BCD) optimization framework with alternating updates between cluster assignments $\mathbf{E}_{S\times C}$ and modality weights $\{\omega(v)\}_{v=1}^V$. The iterative scheme proceeds as follows:

Firstly, given current cluster indicators $\mathbf{E}^{(t)}$, the analytical solution of $\{\omega^{(t+1)}(v)\}_{v=1}^V$ can be obtained as Eq. (17).

$$\begin{gathered} \omega^{(t+1)}(v) = \frac{\sum_{v'=1}^V \sqrt{\epsilon^{(t+1)}(v')}}{\sqrt{\epsilon^{(t+1)}(v)}}, \forall v \\ \epsilon^{(t+1)}(v) = \sum_{p=1}^C \|\mathbf{L}(v)\mathbf{e}_p^{(t)}\|_1. \end{gathered} \tag{17}$$

Secondly, fix $\{\omega(v)\}_{v=1}^{V}$ and update $\mathbf{E}_{S\times C}$. We define the matrix $\widehat{\mathbf{L}}$ as Eq. (18).

$$\widehat{\mathbf{L}} = \sum_{v=1}^{V} \omega(v)(\mathbf{L}(v) + (\mathbf{L}(v))^T). \tag{18}$$

Meanwhile, define $\mathbf{e}_{1\times C}^{[i]}$ as the row vector where the i-th element is 1 and the remaining elements are 0, $\mathbf{e}_{1\times C}^{[0]}$ as a zero vector, and $\mathbf{E}^{[\mathbf{i}]}$ as a matrix with $\mathbf{e}^{[i]}$ as the d-th row and the remaining rows being the same as $\mathbf{E}$.

Next, we traverse the number of rows d and update the d-th row. Our objective function is:

$$y = \underset{i\in\mathbb{Z}_{[1,C]}}{\operatorname{argmin}} \sum_{p=1}^{C} (\mathbf{e}_p^{[i]})^T \widehat{\mathbf{L}} \mathbf{e}_p^{[i]}. \tag{19}$$

If we define:

$$\mathcal{L}_{i,p} = (\mathbf{e}_p^{[i]})^T \widehat{\mathbf{L}} \mathbf{e}_p^{[i]}, i \in \mathbb{Z}_{[0,C]}, p \in \mathbb{Z}_{[1,C]}, \tag{20}$$

then the objective function is transformed into:

$$y = \underset{i\in\mathbb{Z}_{[1,C]}}{\operatorname{argmin}} \mathcal{L}_{i,i} - \mathcal{L}_{0,i}. \tag{21}$$

Find the index in the d-th row vector $\mathbf{e}^d$ where the element is 1 and denote it as j. Then, calculate $\mathcal{L}_{i,i} - \mathcal{L}_{0,i}$ based on Eq. (22).

$$\mathcal{L}_{i,i} - \mathcal{L}_{0,i} = \begin{cases} 2\mathbf{e}_i^T \widehat{\mathbf{l}}_d - \widehat{l}_{d,d}, & \text{If } i = j, \\ 2\mathbf{e}_i^T \widehat{\mathbf{l}}_d + \widehat{l}_{d,d}, & \text{If } i \neq j. \end{cases} \tag{22}$$

When traversing all $\mathcal{L}_{i,i} - \mathcal{L}_{0,i}$ corresponding to i, the optimal solution y can be found, that is, the optimal solution in row d:

$$\bar{\mathbf{e}}^d = \delta(y, C), \tag{23}$$

where $\delta(y, C)$ is a row vector where the y-th element is 1 and the remaining elements are 0.

Alternate update $\mathbf{E}_{S\times C}$ and $\{\omega(v)\}_{v=1}^{V}$ until convergence. The terminal cluster assignments are obtained by mapping $\mathbf{E}$ to supercell $\{SC(s)\}_{s=1}^{S}$.

2.2 Sensitivity analyses for key parameters

2.2.1 Sensitivity analyses on different cluster numbers

We conducted systematic evaluations of clustering sensitivity across benchmark datasets by measuring **ARI/NMI** variation with cluster number (Figs. 12-13). On four datasets (PBMC_Inhouse, Sim, mESC, PBMC_Cao), scHG achieves exact alignment between algorithm-optimized (green) and ground-truth (yellow) cluster numbers, with identical **ARI/NMI** rankings. For the remaining two datasets (PBMC10×, Bmcite), while minor discrepancies existed in cluster number estimation, our model achieved indistinguishable performance compared to ground-truth configurations.

Across half of the benchmark datasets (PBMC10×, Bmcite, PBMC_Cao), the top-10 thresholds achieve 84.6–99.6% of the maximum **ARI** and 80.0–98.3% of the maximum **NMI**, indicating high robustness to cluster number selection.

For the remaining datasets (PBMC_Inhouse, Sim, mESC), the top-10 thresholds achieve 36.6–58.5% of the maximum **ARI** and 40.6–61.2% of the maximum **NMI**, while maintaining 100% accuracy in cluster number estimation (Fig. 12–13), underscoring the method's capability to precisely determine cluster numbers across sensitivity ranges.

Algorithm 1 The Algorithm of scHG

Require: $\{\mathbf{X}(v)\}_{v=1}^{V}$
Ensure: $\{\omega(v)\}_{v=1}^{V}, \mathbf{E}$
Calculate $\{\mathbf{A}(v)\}_{v=1}^{V}$ via (3).
Calculate $\{SC(s)\}_{s=1}^{S}$ via (8) to (12).
Calculate $\{\mathbf{AS}(v) \in \mathbb{R}^{S\times S}\}_{v=1}^{V}$ via (14).
Initialize $\mathbf{E}$.
while not converge **do**
    update $\{\omega(v)\}_{v=1}^{V}$ via (17).
    Calculate $\widehat{\mathbf{L}}$ via (18).
    while not converge and $d \in [1, S]$ **do**
        Find the index of 1 in ed and save as j.
        Calculate $y = \underset{i\in\mathbb{Z}_{[1,C]}}{\operatorname{argmin}} \mathcal{L}_{i,i} - \mathcal{L}_{0,i}$ via (22) and get $\bar{\mathbf{e}}^d = \delta(y, C)$.
        $d \Leftarrow d + 1$
    end while
end while

2.2.2 Sensitivity analysis of hyperparameters α and β

During supercell construction, two hyperparameters, α and β, are introduced to regulate the aggregation process. To systematically examine their impact on clustering performance, we performed a grid-based hyperparameter analysis in which α was varied from 1 to 10 and β was varied from 1 to α. The clustering performance obtained under different (α, β) configurations is visualized as a three-dimensional bar plot in Fig. 14-15, facilitating a comprehensive evaluation of performance trends and parameter sensitivity across the explored hyperparameter space.

As shown in Fig. 14-15, clustering performance remains largely stable across the explored (α, β) hyperparameter space on all six datasets, with minor deviations observed only under a small number of imbalanced parameter settings. This observation indicates that scHG is not overly sensitive to precise hyperparameter tuning and exhibits strong robustness across a wide range of parameter settings, thereby reducing the risk of performance degradation in practical applications.

2.2.3 Sensitivity analysis of similarity threshold γ

During the construction of the cell similarity matrix $\mathbf{A}(v)$, the neighborhood of each cell was first identified by extracting the top $(\gamma + 1)$ most correlated cells (excluding self) from matrix $\mathbf{PCC}(v)$, based on which $\mathbf{A}(v)$ was subsequently computed. To further examine the effect of the neighborhood size parameter γ on clustering performance, we systematically varied γ from 6 to 25 (resulting in 20 distinct settings). Clustering performance was evaluated using ARI and NMI for each configuration, and the corresponding results are presented as line plots in Fig. 16, enabling a clear assessment of performance trends and parameter sensitivity.

Fig. 16 illustrates the effect of the neighborhood size parameter γ on clustering performance across six datasets. On the PBMC10×, PBMC_Inhouse, Bmcite and PBMC_Cao datasets, both ARI and NMI vary smoothly as γ changes, indicating that clustering performance is largely insensitive to moderate variations in neighborhood size. In contrast, more evident performance fluctuations are observed on the Sim, and mESC datasets, suggesting a higher sensitivity to the choice of γ in these cases.

Across all datasets, a consistent empirical tendency can be observed: values of γ associated with relatively stronger clustering performance are closely related to the

dimensionality of the omics features used for clustering. Specifically, when the maximum number of features in the employed omics dataset is below 5000, setting $\gamma = 10$ generally yields more favorable clustering results, whereas $\gamma = 20$ tends to perform better for datasets with higher feature dimensionality. This empirical tendency motivates the choice of γ in scHG configuration, as described in Section "Hyperparameter configuration".

2.2.4 Sensitivity analysis of supercell construction

During supercell construction, random pruning is introduced. Unless otherwise stated, all results reported were obtained using a fixed random seed, ensuring full reproducibility. To explicitly assess the impact of the introduced randomness, we performed 20 independent clustering runs on each of the six datasets, and summarize the resulting performance statistics in Table A.

Table A. Randomness Analysis of scHG Across Six Datasets (Mean ± Variance over 20 Runs)

Metrics \ Datasets	PBMC10×	PBMC_Inhouse	Sim	Bmcite	mESC	PBMC_Cao
**ARI**	**0.8774**±0.0000	**0.7029**±0.0000	**0.9646**±0.0000	**0.6732**±0.0000	**0.9373**±0.0000	**0.5299**±0.0000
**NMI**	**0.8230**±0.0000	**0.7992**±0.0000	**0.9598**±0.0000	**0.6917**±0.0000	**0.8589**±0.0000	**0.5972**±0.0000

For both clustering metrics (ARI and NMI), the observed variance is consistently zero across all datasets, indicating that scHG yields identical clustering outcomes across repeated runs. This result suggests that the randomness introduced during supercell construction does not propagate to the final clustering results and exhibits strong robustness to stochastic operations.

2.3 A summary table of all hyperparameters and default values

Table B. Summary of hyperparameters and default values.

**Parameter**	**Symbol**	**Default**
Adaptive regularization	γ	$\begin{cases} 10, & \max_v\{m_v\} < 5000 \\ 20, & \text{otherwise} \end{cases}$
Second-order parameter	α	5
Second-order parameter	β	2
RNA weight	–	$\frac{2}{9}$
ADT/ATAC weight	–	$\frac{3}{9}$
Fused-feature weight	–	$\frac{4}{9}$
Cluster number estimator	C	$\text{round}\left(\frac{2}{9}C^{(1)} + \frac{3}{9}C^{(2)} + \frac{4}{9}C^{(\tilde{\mathbf{A}})}\right)$

Usage. γ is used in the construction of the similarity matrix $\mathbf{A}^{(v)}$ (Eq. (3)); α and β are used in the second-order co-occurrence matrix $\mathbf{N}^{(v)}$ (Eq. (8)); the weights $\frac{2}{9}$, $\frac{3}{9}$, and $\frac{4}{9}$ correspond to the contributions of RNA, ADT/ATAC, and fused similarity features, respectively, in the cluster number estimation formula; and C denotes the final fused estimate of the cluster number.
